# Tumor-Like Stem Cells Derived from Human Keloid Are Governed by the Inflammatory Niche Driven by IL-17/IL-6 Axis

**DOI:** 10.1371/journal.pone.0007798

**Published:** 2009-11-11

**Authors:** Qunzhou Zhang, Takayoshi Yamaza, A. Paul Kelly, Shihong Shi, Songlin Wang, Jimmy Brown, Lina Wang, Samuel W. French, Songtao Shi, Anh D. Le

**Affiliations:** 1 Center for Craniofacial Molecular Biology, School of Dentistry, University of Southern California, Los Angeles, California, United States of America; 2 Department of Dermatology, King-Harbor Medical Center, Los Angeles, California, United States of America; 3 Salivary Gland Disease Center and the Molecular Laboratory for Gene Therapy, Capital Medical University School of Stomatology, Beijing, China; 4 Department of Otolaryngology, Head & Neck Surgery, Stanford University School of Medicine, Stanford, California, United States of America; 5 Department of Pathology, Kenneth Norris Jr. Cancer Center, University of Southern California Keck School of Medicine, Los Angeles, California, United States of America; 6 Department of Pathology, Harbor-UCLA Medical Center, Torrance, California, United States of America; Harvard Institute of Medicine, United States of America

## Abstract

**Background:**

Alterations in the stem cell niche are likely to contribute to tumorigenesis; however, the concept of niche promoted benign tumor growth remains to be explored. Here we use keloid, an exuberant fibroproliferative dermal growth unique to human skin, as a model to characterize benign tumor-like stem cells and delineate the role of their “pathological” niche in the development of the benign tumor.

**Methods and Findings:**

Subclonal assay, flow cytometric and multipotent differentiation analyses demonstrate that keloid contains a new population of stem cells, named keloid derived precursor cells (KPCs), which exhibit clonogenicity, self-renewal, distinct embryonic and mesenchymal stem cell surface markers, and multipotent differentiation. KPCs display elevated telomerase activity and an inherently upregulated proliferation capability as compared to their peripheral normal skin counterparts. A robust elevation of IL-6 and IL-17 expression in keloid is confirmed by cytokine array, western blot and ELISA analyses. The altered biological functions are tightly regulated by the inflammatory niche mediated by an autocrine/paracrine cytokine IL-17/IL-6 axis. Utilizing KPCs transplanted subcutaneously in immunocompromised mice we generate for the first time a human keloid-like tumor model that is driven by the *in vivo* inflammatory niche and allows testing of the anti-tumor therapeutic effect of antibodies targeting distinct niche components, specifically IL-6 and IL-17.

**Conclusions/Significance:**

These findings support our hypothesis that the altered niche in keloids, predominantly inflammatory, contributes to the acquirement of a benign tumor-like stem cell phenotype of KPCs characterized by the uncontrolled self-renewal and increased proliferation, supporting the rationale for *in vivo* modification of the “pathological” stem cell niche as a novel therapy for keloid and other mesenchymal benign tumors.

## Introduction

Keloid is an exuberant scar unique to human with highest incidence in African-Americans, resembles benign tumor behavior by its aggressive dermal growth that continues to expand beyond the boundaries of the original wound margins (Figure S1A), rarely regresses as observed in hypertrophic scars [1], and recurs at a high rate after surgical removal [2]. A more tolerable and effective modality for keloid treatment is intralesional steroid injection, however, not applicable to very large or multiple keloids [3]. These dermal growths, similar to other fibroid tumors, could potentially reach a grotesque size in the craniofacial region with tremendous esthetic, functional, and psychologically debilitating sequelae [1]. As a chronic inflammatory and fibro-proliferative disease (Figure S1A), keloids exhibit distinctive histological features including a high density of mesenchymal cells, an abundant ECM stroma characterized of whorls of irregularly oriented and thickened hyalinized collagen bundles that are classically described as keloidal collagen [4], a local infiltration of inflammatory cells including mast cells and lymphocytes, and a milieu of enriched cytokines, especially transforming growth factor-β1 (TGF-β1) and IL-6 [5], [6]. Clinically, keloids have been correlated with some degree of inflammatory response associated with tissue injury and most often, manifested clinically as a tender, painful, pruritic or burning sensation. We postulate that the altered or “pathological niche” driven by the inflammatory cytokine IL-17/IL-6 axis potentially regulates the benign, yet aggressive growth behavior of the dermal tumor. A recent study suggests that the interaction between stem cells and keloid-derived fibroblasts may contribute to keloid formation by triggering progressive myofibroblastic differentiation and secretion of abundant extracellular matrix [7]. In addition, the fibroproliferative nature of keloids suggests that mesenchymal stem cells may serve as primary source of multipotent cells to rapidly repopulate the wound site in response to exogeneous insults, such as trauma, surgical injury, or infection. Therefore, keloids provide an ideal benign tumor model to study the role of stem cells and their special niche components in promoting and maintaining the proliferative growth of the dermal scar.

The fate of the stem cells, undergoing self-renewal or differentiation, is dependent on a specialized microenvironment or niche in which the cells reside [8]. The stem cell niche [9] encompasses all elements immediately surrounding the stem cells, including non-stem cells, the extracellular matrix (ECM), as well as soluble molecules present in the locale [10], [11]. Under homeostatic or physiological conditions, the extrinsic niche components or growth factors protect the stem cells from excessive proliferation by providing a balanced proliferation-inhibiting and proliferation-promoting signal [12], [13]. Meanwhile, the stem cells must periodically activate to produce specific lineage progenies for the regeneration or repair of tissues [10]. Therefore, the maintenance of a steady state between stem cell quiescence and activity is the hallmark of a functionally normal niche [13], whereas the deregulation of niche signals may lead to the uncontrolled self-renewal and proliferation of the stem cells, thus contributing to the emergence of the so-called niche-generated diseases including pre-cancer and tumorigenesis [11], [14].

Interleukin (IL)-6 is produced at the site of acute inflammation [15] and plays a critical role in both cellular and humoral immune responses [16]. IL-6 can trigger the switch from acute to chronic inflammation by enhancing the recruitment of monocytes [17], thereby, serving as a key player in both acute and chronic inflammation [18]. Numerous studies have reported elevated IL-6 levels in several inflammatory diseases, including chronic inflammatory and fibro-proliferative diseases such as keloids [5], [6], rheumatoid arthritis, inflammatory bowel disease (colitis), multiple sclerosis, and pulmonary fibrosis [19], [20]. Aside from its established function as an immuno-modulator, IL-6 may contribute to the regulation of stem cell functions via several pathways leading to the inhibition of lymphocyte apoptosis [21] and the maintenance of hematopoietic stem cells [22]. Most recently, IL-6 has been reported as a critical tumor promoter during early colitis-associated tumorigenesis [23], [24]. Immune cells, which often infiltrate tumor and pre-neoplastic lesions, are capable of perpetuating a localized inflammatory response via a variety of cytokines and chemokines, and enhancing the growth and survival of premalignant cells. IL-6 has also been demonstrated to trigger malignant features in Notch-3-expressing stem/progenitor cells from human ductal breast carcinoma and normal mammary gland [25] and further up-regulates telomerase activity in human malignancies [26], [27]. Since telomerase plays an essential role in the regulation of cell proliferation and senescence [28], [29] it has been routinely used as a functional marker to assess stem cell function and tissue homeostasis [30], [31].

IL-17, a recently discovered pro-inflammatory cytokine, is secreted by a distinct subtype of activated CD4^+^ T-cells known as Th17 [32], [33]. Most recently, several studies have clearly demonstrated the critical role of TGF-β and IL-6 or other inflammatory cytokines in the differentiation of human Th17 cells [34]–[37]. The finding of this cytokine has changed our perspectives on chronic inflammatory diseases, including inflammatory bowel disease, rheumatoid arthritis, psoriasis, multiple sclerosis, and allergic skin immune responses [38]–[41]. IL-17 is produced exclusively by activated T cells, however, its receptor is ubiquitously expressed in many cell types, thus making them potential targets [35], [42]. IL-17 amplifies the immune response mediated by a variety of cytokines such as IL-6, tumor necrosis factor (TNF)- α and IL-1β, chemokines such as monocyte chemoattachment protein-1 (MCP-1) and macrophage inflammatory protein-2 (MIP-2)/IL-8, cell-surface markers such as intercellular adhesion molecule-1 (ICAM-1), and pro-inflammatory mediators including prostaglandin E2, nitric oxide, cyclooxygenase-2, and C-reactive protein [43]–[47]. Among these inflammatory cytokines, IL-6 seems to be a major IL-17 signaling target in a variety of cells including macrophages, fibroblasts, osteoblasts, epithelial cells, and chondrocytes [44], [45], [48]–[51]. More importantly, IL-6 not only functions downstream of IL-17 but also acts as a critical upstream target of IL-17, thus forming a paracrine/autocrine feedback loop that promotes autoimmune and allergic diseases [50], [51]. In addition to its immuno-regulatory role, IL-17A promotes the proliferation, migration, and osteogenic differentiation of human bone marrow mesenchymal stem cells (BMMSCs) [52]. Based on existing findings we asked whether IL-17 and IL-6 coordinately interact and contribute to the persistent local tissue inflammation in keloid, i.e. sustaining the “pathological” niche, therefore support the proliferation-promoting signal of the benign dermal growth.

Previously, stem cells derived from the dermis have been isolated and cultured under either adherent conditions with fetal bovine serum (FBS)-containing medium [53] or neural sphere-forming condition in serum free medium supplemented with epidermal growth factor (EGF) and fibroblast growth factor (FGF)-2 [54], [55], [56]. In the current study, using both adherent and sphere-culturing conditions we have isolated and characterized a population of adult stem cells from the dermal layer of keloid scars, named keloid derived precursor cells (KPCs), which are characterized by several defined stem cell features including clonogenicity, self-renewal, expression of distinct embryonic and mesenchymal stem cell markers, and multipotent differentiation. We have also defined an essential inflammatory cytokine paracrine loop between IL-17 and IL-6, constituting the “pathological” keloid niche that is capable of elevating the proliferation-promoting signal. Using KPCs transplanted in immunocompromised mice we generate a keloid-like tumor model that is driven by the *in vivo* inflammatory niche. Understanding how the inflammatory niche regulates tumor-like stem cell growth in keloid is critical in further elucidation of keloid pathogenesis and will open a new avenue for benign tumor stem cell research and ultimately, keloid therapies.

## Materials and Methods

### Ethics Statement

This study followed the tenets of the Declaration of Helsinki and all keloid tissues were obtained as discarded biological samples from patient donors (aged 20∼50 years of age) after a written informed consent was provided. This study was approved by the Institutional Ethics Committee/Institutional Review Board (IRB) of both University of Southern California and Harbor-UCLA/King Drew Medical Center.

### Antibodies and reagents

All antibodies used in this study were listed in Table S1. Recombinant human interleukin-6 and interleukin-17A were purchased from *PeproTech* Inc. (Rocky Hills, NJ, USA). All chemical reagents were analytical grade and obtained from Sigma-Aldrich (St. Louis, MO).

### Animals

C57BL/6 mice were purchased from Jackson Lab. Immunocompromised mice (bg-nu/nu-xid) were purchased from Harlan Sprague Dawley Inc. (Indianapolis, IN) and were used for *in vivo* transplantation experiments under the approved animal protocol of University of Southern California (USC #10874).

### Cell isolation and culture

Keloid and matched peripheral normal skin tissues were obtained from the same patient donor and were used for isolation of stem cells. At least 5 sets of keloid derived progenitor cells (KPCs) with appropriately matched skin derived progenitor cells (SKPs) were derived from 5 unrelated patients [(3 African-Americans (2 females, 1 male), 2 Hispanics (1 female, 1 male)], and were used in all experiments described. SKPs were also derived from normal skin tissues from other donors. Tissues were treated aseptically, cut into 5–6 mm pieces, and incubated in sterile phosphate-buffered saline solution (PBS) containing 3 mg/mL dispase (Sigma) overnight at 4°C. The epidermis was manually stripped off; the dermal portion was minced into 1-mm^3^ pieces, digested in sterile PBS containing 4 mg/mL collagenase I (Worthington Biochemical Corporation, Lakewood, NJ) for 2 hours at 37°C [54], [57], filtered through a 70 µm cell strainer (Falcon, Franklin Lakes, NJ), and the isolated cells were cultured under two different conditions. For sphere-forming culture [54], [55], [56], cells were seeded on uncoated dishes with growth medium, Dulbecco's minimum essential medium with low glucose (DMEM-LG)/F12 (3∶1) (Invitrogen, Carlsbad, CA) supplemented with 40 ng/ml fibroblast growth factor (FGF-2), 20 ng/ml epidermal growth factor (EGF) (Chemicon, Billerica, MA), B27, 1 µg/ml fungizone and 100 U/ml penicillin/100 µg/ml streptomycin (Invitrogen). After 2∼4 weeks, spheres were formed and further sub-cultured in 60 mm ultra-low culture dishes (Corning Inc., Corning, NY). For cell attachment or MSC culture, cells were cultured as described previously with some modifications [53]. Briefly, resuspended cells were plated on non-treated 10-cm Petri dishes (VWR Scientific Products, Willard, OH) with minimum essential medium (MEM: Invitrogen) containing 10% fetal bovine serum (FBS: Clontech Laboratories, Inc., Mountain View, CA), 100 U/ml penicillin, 100 µg/ml streptomycin (Invitrogen), 2 mM L-glutamine, 100 mM non-essential amino acid (NEAA), and 550 µM 2-mercaptoethanol (2-ME; Sigma-Aldrich), and cultured at 37°C in a humidified tissue-culture incubator with 5% CO_2_ and 95% O_2_. The confluent cells were passaged with 0.05% trypsin containing 1 mM EDTA, and continuously subcultured in the complete growth medium.

### Colony forming unit fibroblasts (CFU-F) assay

The CFU-F assay was performed as previously described [58], [59]. Cell aggregates containing more than 50 cells were counted as colonies using a dissecting microscope. The CFU-F assay was repeated in 5 independent experiments.

### Single cell cloning

Precursor cells isolated from keloid tissues and matched peripheral normal skin samples of different donors were seeded in non-coated tissue culture 96-well plates (Falcon) at a concentration of 2 cells/ml (200 µl/well, at least 4 plates/donor). The plates were screened for presence of single cell colony while wells contained more than two colonies were excluded from further analysis. Wells containing a single cell were allowed to reach confluency, transferred to 24-well dishes, and further expanded in the growth medium [57].

### Population doubling assay

Clonal dermal precursor cells at each passage (P2, P5, P10 and P20) were seeded at 1.0×10^3^ cells in 35-mm dishes in MSC medium for each period (0, 2, 4, 6, 8, 10 days). Cells were suspended with 0.05% trypsin-EDTA and cell number was determined by hemacytometer. Population doubling time (PDT) was calculated with the formula, PDT = (t-t_0_)_*_lg2/lg(N/N_0_) (N_0_ and N represent the cell numbers at time t_0_ and t, respectively). Meanwhile, the accumulated population doublings were determined and calculated according to the standard 3T3 protocol as described previously [60].

### Multipotent differentiation

We tested the *in vitro* multi-differentiation capabilities of dermal precursor cells including osteogenesis, adipogenesis and neural cell differentiation as described in Methods S1.

### Stimulation of cells with IL-6 or IL-17

KPCs and SKPs at 70∼80% confluence were serum-starved (medium containing 1% FBS) for 24 hours followed by exposure to different concentrations of IL-6 or IL-17 (R & D Systems). Under certain conditions, to clarify the role of IL-17/IL6 axis in the regulation of KPC functions, the serum-starved cells were pretreated with different concentrations of specific neutralizing antibodies for human IL-6 or IL-17 or an isotype-matched control IgG (R & D Systems) for 1 hour followed by stimulation with IL-6 or IL-17 for 24 hours. Then whole cell lysates or total RNA were extracted and subjected to Western blot and RT-PCR analysis, respectively.

### Cell proliferation assay

The proliferation of dermal precursor cells was assessed by 5-bromo-2-deoxyuridine (BrdU) incorporation using a BrdU staining kit (Invitrogen) as previously described [55]. To quantify cell proliferation capacity, ten representative images were used to enumerate BrdU-positive cells and quantitated as a percentage of BrdU-positive cells over total nucleated cells.

### Flow cytometric analysis

Approximately 5×10^5^ cells were incubated with phycoerythrin (PE)- or fluorescein isothiocyanate (FITC)-conjugated mouse monoclonal antibodies specific for human CD34, CD45, CD105, CD146, CD29, CD73, CD90, and Stro-1, or incubated with purified mouse monoclonal antibodies specific for human IL-6 receptor (IL-6R) and IL-17 receptor (IL-17R) followed by incubation with FITC-conjugated secondary antibodies (Table S1). The labeled cells were subjected to Epics® XL-MCL Beckman Coulter flow cytometer, and analyzed using the Expo32 program (USC Clinical Pathology Reference Lab). Cell samples incubated in the absence of primary antibody or with an isotype-matched control IgGs (Southern Biotech, Birmingham, AL) were used as negative controls [58], [59].

### Reverse transcription–polymerase chain reaction (RT-PCR) and quantitative real-time PCR (qPCR)

Total RNA was isolated from cultured cells using TRIZOL^®^ Reagent (Invitrogen). RT-PCR was carried out using the One-step RT-PCR Kit (QIAGEN, Valencia, CA), whereby the number of amplification cycles and the amplifying condition for individual target genes were determined to be in the linear range. To confirm RT-PCR results in some experiments, qPCR was performed using the iScript one-step RT-PCR kit with SYBR Green (BioRad, Hercules, CA) according to the manufacturer's instructions. As an internal control, levels of β-actin were determined in parallel with the target genes. All primers (Table S2) were synthesized at the Core Facility at Norris Comprehensive Cancer Center, USC.

### Western blotting

We performed electrophoresis of protein extracts prepared from cultured cells or tissue lysates followed by immunoblotting as previously described [57]. Blots were incubated with primary antibodies at a dilution of 1∶1000∼1∶2000 followed by horseradish peroxidase (HRP)-conjugated secondary antibodies (PIERCE, Rockford, IL), and visualized by the enhanced chemiluminescence light method (PIERCE). For standardization, blots were re-probed with mouse antibody to human β-actin.

### Cytokine antibody array

To analyze the cytokine expression profile of tissue lysates we utilized the RayBio Human Cytokine Antibody Array 3 (RayBiotech, Inc., Norcross, GA), which allows the detection of 42 cytokines, chemokines and growth factors in one experiment following manufacturer's protocol.

### Telomerase activity and telomeric length assay

The telomerase activity was measured using the telomere repeat amplification protocol (TRAP) TeloTAGGG telomerase PCR ELISA kit (Roche Diagnostics, Indianapolis, IN). The telomerase-mediated elongated product was detected by hybridization to digoxigenin-labeled probes and enzyme activity quantified by photometric enzyme immunoassay.

### Enzyme-linked immunoassay (ELISA)

The level of IL-6 and IL-17 in tissue and cell lysates was detected using human IL-6 ELISA Ready-SET-Go (eBioscience, San Diego, CA) and human or mouse IL-17 Quantikine ELISA Kit (R&D Systems, Minneapolis, MN), respectively, following the manufacturers' instructions.

### *In vivo* transplantation

Transplantation studies were carried out using KPCs and matched SKPs derived from five different donors, and each transplantation was done in triplicates (n = 3). Approximately 2.0×10^6^ stem cells mixed with 40 mg of hydroxyapatite/tricalcium phosphate (HA/TCP) ceramic powder (Zimmer Inc., Warsaw, IN), or Gelfoam (3 mm×3 mm×2 mm, Pharmacia, Piscataway, NJ), were subcutaneously transplanted into the dorsal surface of 8–10-week-old female immunocompromised mice as previously described [55], [56]. Serial transplantation was described in Methods S. For the hydrogel carrier transplant, we mixed 2.0×10^6^ cells with Heprasil™ (CMHA-S, thiol-modified hyaluronic acid with thiol-modified heparin) and Gelin-S™ (GTN-DTPH, thiol-modified gelatin) (1∶1) with or without IL-6, and then combined with Extralink™ (PEGDA, polyethylene glycol diacrylate) (4∶1) (Glycosan Biosystems, Salt Lake City, UT). The immobilized heparin in Heprasil™ mimics the heparan sulfate proteoglycans of the extracellular matrix with increased avidity for growth factors by forming non-covalent binding to the polymer network, prevents proteolysis, and maintains their bioactive state with a slow release over several weeks (Glycosan Biosystems) [61], [62]. The mixtures were immediately injected subcutaneously into the dorsal surface of 8–10-week-old female immunocompromised mice.

To further investigate the role of IL-6 in the initiation of KPC-generated transplants, KPCs mixed with hydrogel with or without human IL-6 neutralizing antibody (10 µg/ml) were injected subcutaneously into immunocompromised mice for 8 weeks. On the other hand, to investigate the role of IL-6 in the maintenance of KPC-generated transplants, KPCs mixed with hydrogel were injected subcutaneously into immunocompromised mice for 4 weeks, followed by intra-lesional injection of IL-6 neutralizing antibody twice a week (5 µg/time) for another 5 weeks. In all cases, an isotype-matched normal mice IgG was used as negative control.

### Adaptation of activated CD4^+^CD25^−^ T-lymphocytes in immunocompromised mice

Spleens were harvested from C57BL/6 mice for isolation of CD4^+^CD25^−^ T-lymphocytes using MidiMACS separator (Miltenyi Biotec) and mouse CD4^+^CD25^+^ T lymphocyte isolation kit (Miltenyi Biotec) following manufacturer's instruction. The purity of the CD4^+^CD25^−^ T cells was >95%. The cells were seeded at 1×10^6^/well on 24-well multi-plates and cultured in RPMI-1640 medium containing 10% FBS, 50 µM 2ME, 10 mM HEPES, 1 mM sodium pyruvate, 1% NEAA, 2 mM L-glutamine, 100 U/ml penicillin and 100 mg/ml streptomycin followed by stimulation with 5 µg/ml plate-bounded anti-CD3 antibody (BD Biosciences) and 2 µg/ml soluble anti-CD28 (BD Biosciences). After three days, activated cells were collected and washed with normal saline [63]. 1×10^6^/mouse of the activated CD4^+^CD25^−^ T lymphocytes were injected into immunocompromised mice through tail vein two days before KPCs mixed with hydrogel with or without IL-6 were transplanted subcutaneously. In parallel, transplants in mice without infusion of T cells were used as controls.

### Histology and immunohistochemistry

Keloid and matched peripheral normal skin tissues, normal skin and normal scar tissues from both African-American and Caucasian patient donors, and transplant samples were fixed with 10% formalin in PBS or embedded in O.C.T. compound (Sakura, Tokyo, Japan) and snap frozen in liquid nitrogen. Procedures for immunohistochemical studies and electron microscopy were described in Methods S1.

### Immunofluorescence studies

4% paraformaldehyde-fixed cultured cells and sections of normal and keloid tissue samples were immunolabeled with specific primary antibodies followed by FITC- and/or PE-conjugated secondary antibodies (BD Biosciences). After the nuclei were stained with 4′, 6-diamidino-2-phenylindole (DAPI) the samples were observed under a confocal microscope. Isotype-matched control antibodies (Invitrogen) were used as negative controls. Positive signals in at least 5 random fields were visualized and counted.

### Statistical analysis

All data are expressed as mean±SEM from at least five independent experiments. Differences between experimental and control groups were analyzed by two-tailed unpaired Student's *t*-test using SPSS. *P-*values less than 0.05 were considered statistically significant.

## Results

### Keloid contains benign tumor-like stem cells

**Figure 1:**
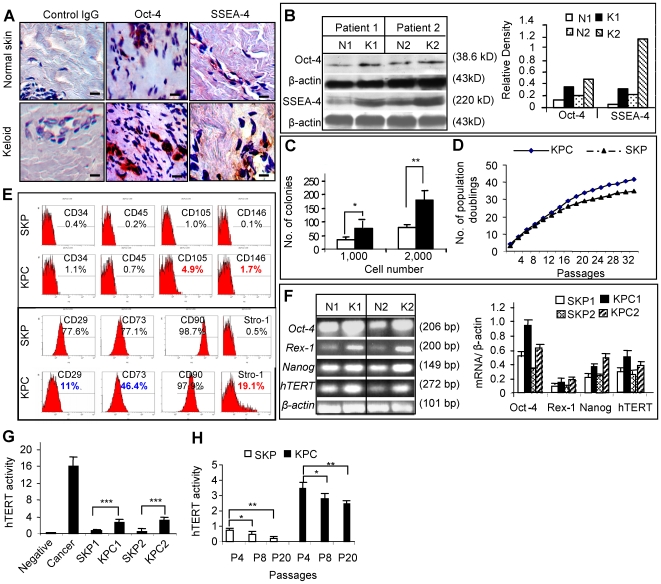
Identification of dermal derived precursor cells from keloid tissues. *A*, Frozen sections of keloid tissues and their matched peripheral normal skins were immunostained with specific antibodies for human Oct-4 and SSEA-4. Scale bars, 50 µm. *B*, Expression of Oct-4 and SSEA-4 in keloids (K1, K2) and matched normal skins (N1, N2) were determined by Western blot analysis and scanning densitometer. *C*, Colony formation analysis of cells derived from keloids and normal skin (mean±SEM). * *P*<0.05; ** *P*<0.01. *D*, Cell population doubling numbers were determined in single cell colony-derived stem cells from keloids (KPCs) and matched peripheral normal skins (SKPs) by standard 3T3 cell culture protocol (mean±SEM). *E*, Flow cytometric analysis of cell surface markers of KPCs and SKPs. *F*, RT-PCR (upper panel) and qPCR (lower panel) analysis of stem cell genes of KPCs (K1, K2) and SKPs (N1, N2). *G* and *H*, Analysis of telomerase activity using Telo*TAGGG* telomerase PCR ELISA kit (mean±SEM). * *P*<0.05; ** *P*<0.01; *** *P*<0.001. Data are representative of at least five independent experiments using specimens obtained from different patient donors with matched normal controls (n = 5).

Aside from the characteristic histological features of abundant ECM stroma, specifically large collagen bundles (Figure S1B), hyper-cellular and inflammatory states, the keloid tissue harbors an elevated level of Octamer-4 (Oct-4) and stage specific embryonic antigen-4 (SSEA-4) positive signals randomly dispersed in the reticular dermis as compared to matched normal skin of the same donor (Figure 1A; Figure S1C). The relatively increased expression of two major markers for embryonic and mesenchymal stem cells [54], [64], [65], Oct-4 and SSEA-4, in keloid tissues as compared to matched normal skin was further confirmed by Western blot (Figure 1B), suggesting that keloid may harbor more postnatal stem cells than normal skin.

Next, we isolated a population of adult stem cells from keloid dermis, named keloid derived precursor cells (KPCs), from 5 independent donors and assess their colony formation and proliferation. Normal skin-derived precursor cells, termed SKPs [54], were derived from matched normal skin of the same donor. Colony formation was observed on day 3 (Figure S1D) in 6∼8% of KPCs whereas only 2∼4% in normal skin precursor cells (SKPs) (Figure 1C), suggesting higher colony-forming capabilities of the tumor-like stem cells in keloids. Single colony formation was further confirmed using diluting seeding density at 2 cells/ml (200 µl per well) (Figure S1E). At early passages (less than passage 5), both KPCs and SKPs exhibited similar doubling time and cumulative population doublings; however, at a later passage, KPCs showed a relatively increased growth rate compared to SKPs, corresponding to an increase in cumulative population doublings and a reduction in doubling time (Figure 1D; Figure S1F). G-banding analysis (Methods S1) demonstrated normal karyotype with no cytogenetic abnormalities in both SKPs and KPCs (Figure S1G), suggesting the maintenance of chromosomal stability in these derived stem cells. These results suggest that keloid-derived stem cells maintain a relatively higher proliferative rate than their normal skin counterparts, and this inherent rapid growth appears unrelated to mutational changes observed in malignant tumor.

Up to date, efforts have been made to develop a cell-surface antigen profile for better purification and identification of mesenchymal stem cells (MSCs) from different tissue sources [10]. There is a consensus that human MSCs rarely express markers of hematopoietic cells such as CD45 and CD34 [10], [64], [66]. Several positive markers such as Stro-1, CD73, and CD106 were considered the most useful and relatively specific markers for MSCs [10], but others including CD90/Thy-1 were relatively non-specific, although frequently expressed on MSCs from different tissues [10], [64], [66]. To characterize stem cell phenotypic markers of KPCs using flow cytometry, we observed that a high percentage of both KPCs and SKPs expressed CD90 at passage 2–6 (Figure 1E). However, only 11% and 46.4% of KPCs expressed CD29 and CD73, respectively, as compared to 77.6% for CD29 and 77.1% for CD73 in SKPs (Figure 1E). Interestingly, the percentage of KPCs expressing Stro-1 (19.1%) and CD105 (4.5%) was consistently higher than that of SKPs (Figure 1E). Similar expression profiles of cell surface molecules were observed in SKPs and KPCs up to 10 passages (data not shown). The differential expression of mesenchymal stem cell marker profiles suggests KPCs are uniquely distinct from SKPs.

We next examined several specific stem cell genes expression using quantitative real-time PCR (qPCR). KPCs expressed relatively higher levels of Oct-4, Rex-1, Nanog, and human telomerase reverse transcriptase (hTERT) mRNAs than SKPs (Figure 1F). In addition, comparative analysis of telomerase activity using *Telo* TAGGG telomerase PCR ELISA showed that KPCs displayed an inherently higher telomerase activity (5–6 folds) as compared to normal skin stem cells (Figure 1G). Unlike cancer, both KPCs and SKPs showed significantly lower level of telomerase activities than cancer cells (Figure 1G), and gradually decreased with increasing cell passages (Figure 1H) suggesting a tighter growth regulation. Taken together, these results suggest that KPCs differ from SKPs in terms of distinct surface marker expression profile, elevated expression of several specific stem cell genes, and increased capability of colony formation and proliferation, thus resembling benign tumor-like properties.

### Keloid-derived precursor cells are capable of multiple differentiation

The multi-differentiation potential of KPCs was determined and compared to that of SKPs. Under adipogenic and osteogenic induction conditions, both SKPs and KPCs could differentiate into adipocytes and osteoblasts as determined by Oil Red O staining (Figure S2A) and by Alizarin Red S staining (Figure S2D), respectively. Adipogenic differentiation was further confirmed by the increased expression of specific adipogenic markers including peroxisome proliferators-activated receptor γ2 (PPARγ2), αP2 and lipoprotein lipase (LPL) as determined by RT-PCR (Figure S2C). Similarly, the osteogenic induction of KPCs and SKPs was further supported by the increased expression of osteocalcin, an osteogenic marker (Figure S2F). In addition, quantification of the Oil Red O and Alizarin Red S (Figures S2B and S2E) staining using 5 random fields as well as RT-PCR results (Figures S2C and S2F) showed a moderate increase in the expression of adipogenic and osteogenic markers in differentiated KPCs compared to differentiated SKPs. However, further evidence is needed to determine whether KPCs have a relatively stronger capability to differentiate toward adipogenesis and osteogenesis as compared to SKPs. Meanwhile, we demonstrated that keloid-derived sphere colonies (Figure S3) were capable of multipotent differentiation into distinct lineages including mesoderm-derived adipocytes, osteoblasts and smooth muscle cell-like cells, and ectoderm-derived different types of neural cells (Figure S3D). These findings consistent with previous stem cell properties described in rodent and mammalian skin-derived precursors or SKPs [54], [55], [56] indicate that keloid derived stem cells may represent an active form of SKPs.

### KPCs are capable of regenerating connective tissues after *in vivo* transplantation

**Figure 2:**
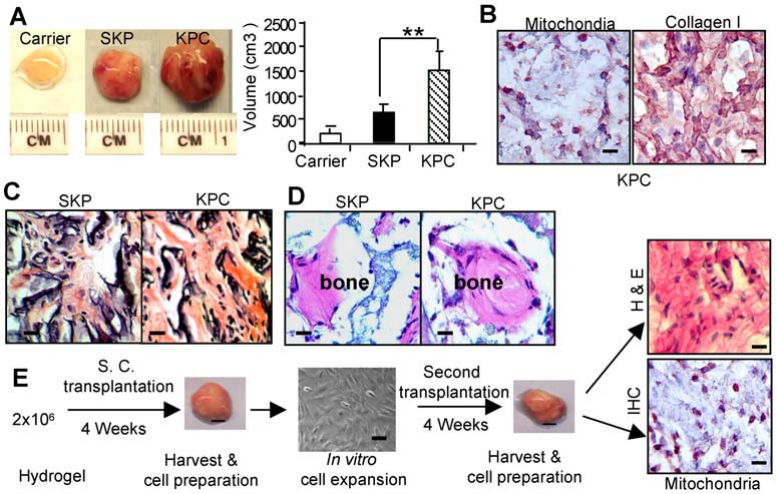
*In vivo* transplantation of dermal stem cells. *A*, Size and volume of transplants generated from SKPs and KPCs using Gelfoam as a carrier for 8 weeks (mean±SEM). ** *P*<0.01. *B*, Immunohistological studies of KPC transplant tissues using a specific antibody for human mitochondria (purple color) and type I collagen (brown color), respectively. Scale bars, 50 µm. *C*, H & E histological stain of transplants. Scale bars, 50 µm. *D*, *In vivo* bone regeneration by SKPs or KPCs using hydroxyapatite/tricalcium phosphate (HA/TCP) as carrier. Scale bars, 50 µm. *E*, Serial transplantation of KPCs. KPCs (2×10^6^) with hydrogel were injected subcutaneously into nude mice. After 4 weeks, the transplanted were harvested and the recovered cells were expanded *in vitro* and re-transplanted into mice for another 4 weeks. The transplant tissues were harvested for H & E staining or immunohistochemical (IHC) staining with a specific antibody for human mitochondria (purple color). Scale bars, 50 µm. The results are representative of five independent experiments.

To explore the *in vivo* regenerative capability, the expanded KPCs and SKPs (2×10^6^) were subcutaneously transplanted with Gelfoam as a carrier in immunocompromised mice. Similar transplants were carried out using human bone marrow mesenchymal stem cells (hBMMSCs) as another source of stem cells. As expected, hBMMSCs were not capable of regenerating tissue up to 8 weeks (data not shown). However, KPCs and SKPs from different donors consistently regenerated connective tissue-like transplants (5 out of 5 mice), with a more rapidly growing tumor-like mass using KPCs as compared to SKPs (Figure 2A). The human origin of cellular components of the transplants was confirmed by immunostaining with specific antibodies for human mitochondria (Figure 2B). Histological features of the transplants resembled early connective tissue phenotype including presence of type I collagen fibrils, however, the characteristic keloid–like thick collagen bundles were not observed (Figures 2B and 2C). Next, to assess the *in vivo* bone regenerative capability, KPCs were implanted subcutaneously with hydroxyapatite/tricalcium phosphate (HA/TCP) as a carrier in immunocompromised mice for 8 weeks. Histological analyses revealed calcified nodules in both KPCs and SKPs generated transplants (Figure 2D).

To further confirm that the isolated KPCs and SKPs are truly stem cells, we carried out a series of transplantation using limiting cell density dilution. As shown in Table S4, KPCs exhibited stronger capability for tumor xenograft formation at a minimal density of 1×10^5^cells after transplantation for 8 weeks as compared to their counterparts, SKPs. We next performed serial transplantation with hydrogel as a carrier using 2×10^6^ KPCs subcutaneously transplanted into immunocompromised mice. At 4 weeks post-primary transplantation, the transplants were harvested and digested single cells were re-transplanted into immunocompromised mice for another 4 weeks. Our results indicated that KPCs recovered from secondary transplants maintained the *in vivo* ability to form connective-like tissues (Figure 2E). These results were unique and particularly striking, as KPCs transplanted to a heterologous environment were capable of proliferating and differentiating into scar-like connective tissues expressing type I collagen. However, we were not successful in replicating the human keloid tumor using the current transplant model, suggesting that KPCs are not the sole contributor to the exuberant scar growth. Therefore, we hypothesize that, aside from KPCs, the environmental niche where KPCs reside, is capable to drive stem cell proliferation and differentiation, and therefore regulates the tumor-like keloid growth.

### Elevated IL-17 and IL-6 coordinately contribute to the unique inflammatory niche of KPCs

**Figure 3:**
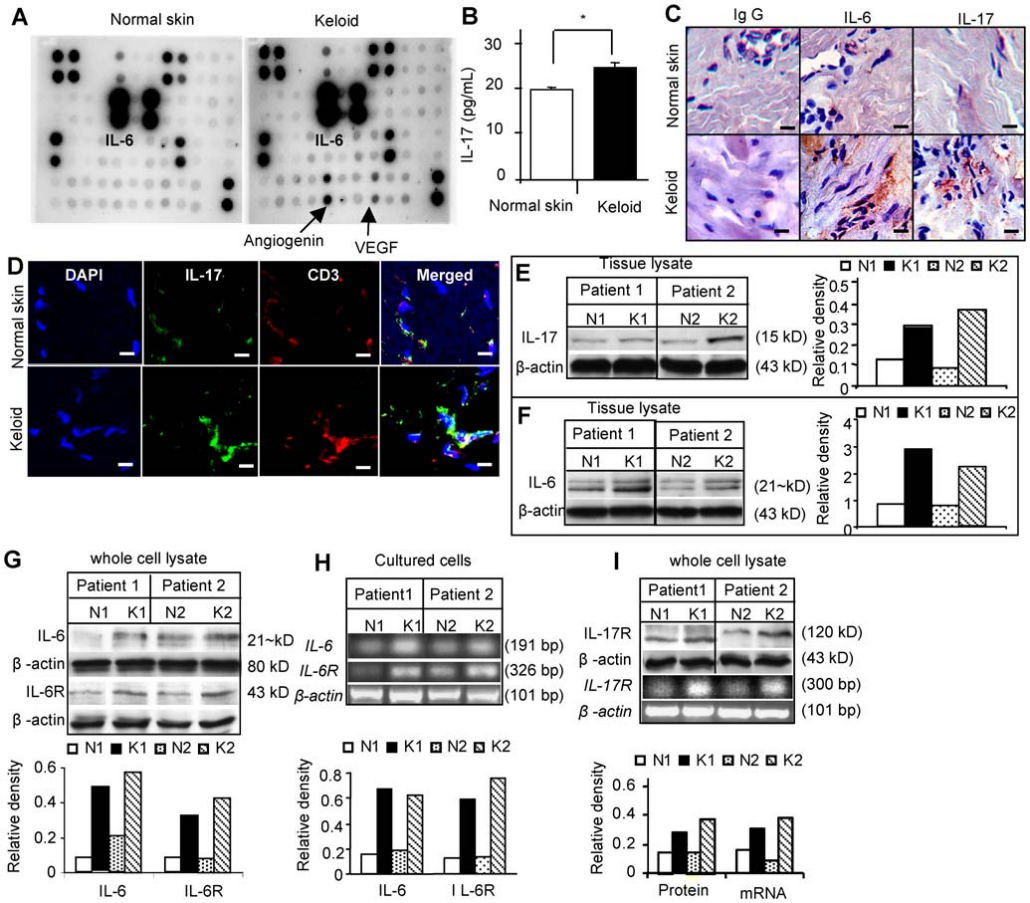
Increased expression of IL-6 and IL-17 in keloids. *A*, Cytokine expression profiling of keloid and matched normal skin tissue lysates by human cytokine antibody array. *B*, Determination of IL-17 levels in keloid and normal skin tissue lysates by ELISA (mean±SEM). * *P*<0.05. *C*, Immunohistochemical studies of IL-6 and IL-17 expression in keloid and matched normal skin tissues. Scale bars, 50 µm. *D*, Confocal immunofluorescence studies show co-localization of IL-17 around CD3^+^ cells as determined by dual-color staining. Scale bars, 100 µm. *E* and *F*, Western analysis of IL-17 (E) and IL-6 expression (F) in keloid tissues and matched normal skins. *G* and *H*, Expression of IL-6 and IL-6 receptor (IL-6R) in precursor cells derived from different keloid samples (K1, K2) and matched normal skins (N1, N2) as determined by Western blot (G) and RT-PCR analyses (H). *I*, Expression of IL-17 receptor (IL-17R) in precursor cells derived from different keloid samples (K1, K2) and matched normal skins (N1, N2) as determined by western blot and RT-PCR analyses. Data are representative of at least five independent experiments using KPCs and matched SKPs from different patient donors (n = 5).

To further explore the concept of niche regulated keloid fibroproliferative growth we next attempted to define the distinct “pathological” niche of keloid by screening a panel of candidate inflammatory cytokines utilizing fresh keloid tissues from different donors. Using a focused human cytokine array we found that the levels of several mediators, including growth-regulated IL-6, oncogene alpha (GROα), IL-1β, MIP-1δ, RANTES, stem cell factor (SCF), TGF-β1, TNF-α, angiogenin II, vascular endothelial growth factor (VEGF) and platelet-derived growth factor beta homodimer (PDGF-BB), were increased by more than 2-folds in keloid tissues as compared to matched normal skin tissues (Figure 3A; Table S3). Particularly, an elevated level of IL-6, a critical cytokine of the T_H_2 cells, and its receptor IL-6R, were detected in all keloid tissues screened and further confirmed by immunohistochemical studies (Figure 3C; Figure S4A) and Western blot analysis (Figure 3F; Figure S4B). Both IL-6 and IL-6R were expressed in SKPs and KPCs, with a relatively higher level in KPCs as compared to the matched SKPs, as determined by Western blot, RT-PCR and flow cytometry (Figures 3G and 3H; Figure S4C).

Another important inflammatory cytokine of the T_H_17 cells [38]–[41], IL-17, was abundantly expressed in keloid tissues as demonstrated by ELISA (Figure 3B) and further confirmed by immunohistochemical staining (Figure 3C) and Western blot analysis (Figure 3E). The increased expression of IL-17 in keloid tissues was discernible around T-cell receptor CD3-positive cells as determined by dual-color immunofluorescence staining (Figure 3D). IL-17 has been reported to be produced almost exclusively by activated T cells, but its receptor is widely expressed in various cell types [42]. Herein, we observed an elevated expression of IL-17R in KPCs as compared to SKPs (Figure 3I; Figure S4C). However, the expression of IL-17 was undetectable in both KPCs and SKPs (data not shown). Taken together, these findings suggest that IL-6 and IL-17 are abundant components of the unique inflammatory niche of keloid and may contribute to the regulation of self-renewal and differentiation of keloid stem cells.

### IL-17 is involved in the regulation of hTERT expression in KPCs via coordinating IL-6 paracrine loop

**Figure 4:**
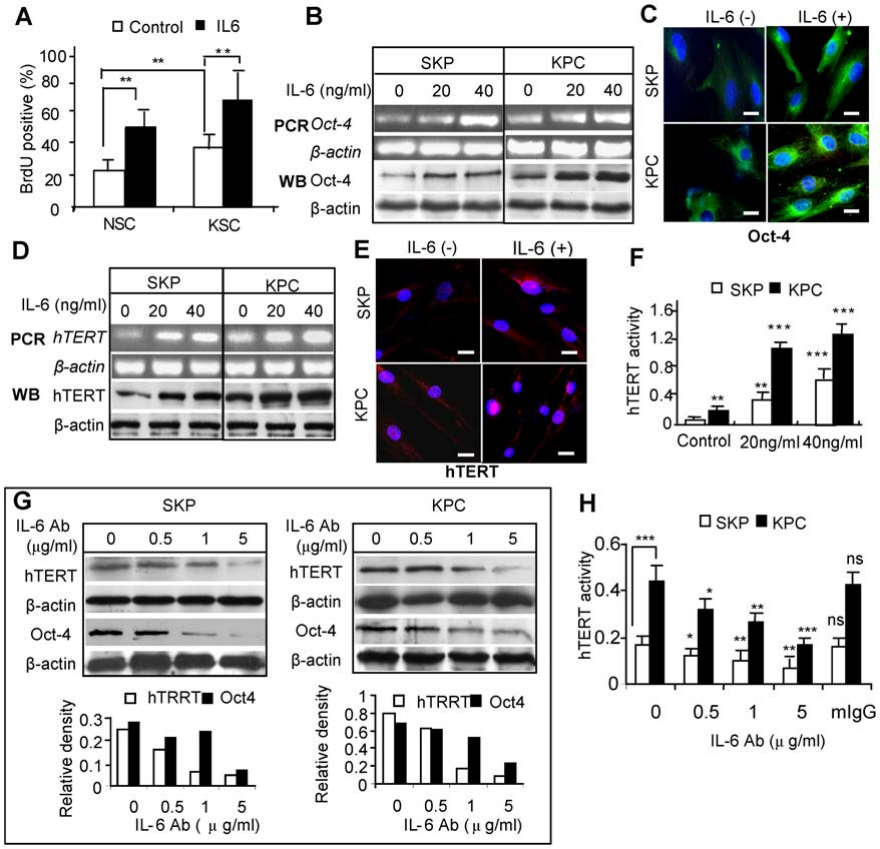
IL-6 increases Oct-4 and telomerase expression in KPCs and SKPs. *A*, KPCs and SKPs were cultured in 1% FBS for 24 hours followed by exposure to different concentrations of IL-6, and BrdU incorporation in KPCs and SKPs was determined (mean±SEM). ** *P*<0.01. *B*, Expression of Oct-4 mRNA and protein were determined by Western blotting (WB) and RT-PCR, respectively. *C*, Immunofluorescence studies of Oct-4 expression in SKP and KPC after incubated with 20 ng/ml IL-6 for 24 hours. Scale bars, 20 µm. *D*, Expression of hTERT mRNA and protein were determined by Western blotting (WB) and RT-PCR, respectively. *E*, Immunofluorescence studies of hTERT expression in SKP and KPC following incubation with 20 ng/ml IL-6 for 24 hours. Scale bars, 20 µm. *F*, Telomerase enzyme activity of SKPs and KPCs in response to IL-6 as determined by Telo*TAGGG* Telomerase PCR ELISA. *G* and *H*, Treatment with neutralizing antibody for IL-6 (IL-6Ab) decreased the basal level of hTERT as determined by Western blotting (G) and Telo*TAGGG* Telomerase PCR ELISA (H). An isotype-matched normal mice IgG (mIgG) was used as negative control (mean±SEM). * *P*<0.05; ** *P*<0.01; *** *P*<0.001; ns, no significance. The results are representative of at least five independent experiments using KPCs and the matched SKPs from different patient donors (n = 5).

IL-6 plays an important role in regulating self-renewal and differentiation of stem cells [21], [22], [25], as well as tumor growth [25]–[27]. Our data showed that IL-6 stimulated BrdU incorporation in both KPCs and SKPs (Figure 4A). Exposure of KPCs and SKPs to exogenous IL-6 resulted in a dose-dependent increase in Oct-4 expression at both mRNA and protein levels (Figures 4B and 4C). In parallel studies, we found that incubation with IL-6 enhanced telomerase (hTERT) expression and enzyme activities in both KPCs and SKPs (Figures 4D, 4E and 4F). The basal level of hTERT was always higher in KPCs as compared to SKPs. Since KPCs constitutively expressed a higher level of IL-6 and IL-6R than SKPs (Figures 3G and 3H; Figure S4C), we next tested whether the intrinsically secreted IL-6 might contribute to the elevated telomerase expression and activity in KPCs. Our data showed that treatment with IL-6 neutralizing antibodies suppressed hTERT expression in both KPCs and SKPs (Figures 4G and 4H) in a dose-dependent manner. However, IL-6 showed no obvious effects on SSEA-4 expression in both SKPs and KPCs as determined by flow cytometry (Figure S5A). Taken together, these data suggest that IL-6 plays a critical role in the regulation of self-renewal and proliferation of KPCs by regulating essential functional genes such as hTERT and Oct-4.

**Figure 5:**
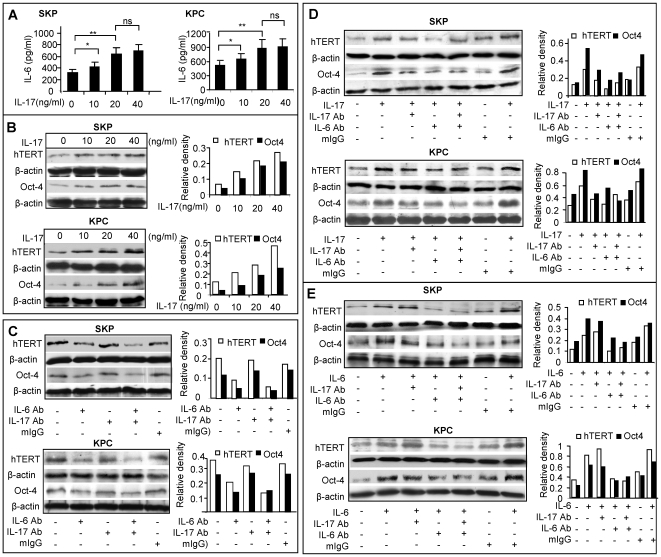
IL-17-induced hTERT and Oct-4 expression is IL-6-dependent. *A*, IL-17 stimulated IL-6 secretion in SKPs and KPCs as determined by ELISA analysis (mean±SEM). ** *P*<0.01; ns, no significance. *B*, IL-17 stimulated the expression of hTERT and Oct-4 in SKPs and KPCs as determined by Western blot analysis. *C*, Blocking IL-6, but not IL-17 attenuated the basal expression of hTERT and Oct-4 in SKPs and KPCs. Cells were treated with 5 µg/mL of neutralizing antibody for either IL-6 (IL-6 Ab), or IL-17 (IL-17Ab), or both for 24 hours and expression of hTERT and Oct-4 was determined by Western blot analysis. *D*, IL-17-induced upregulation of hTERT and Oct-4 expression in SKPs and KPCs was significantly attenuated by treatment with neutralizing antibodies for either IL-17, or IL-6, or both. After serum-starved for 24 hours, cells were pretreated with neutralizing antibodies, followed by incubation with 20 ng/mL of IL-17. The expression of hTERT and Oct-4 was determined by Western blot analysis. *E*, IL-6-induced upregulation of hTERT and Oct-4 expression in SKPs and KPCs was attenuated only by IL-6Ab, not by IL-17Ab. Following serum-starvation for 24 hours, cells were pretreated with neutralizing antibodies and incubated with 20 ng/mL of IL-6. Expression of hTERT and Oct-4 was determined by Western blot analysis. An isotype-matched normal mouse IgG (mIgG) was used as negative controls. The results are representative of at least five independent experiments using KPCs and the matched SKPs from different patient donors (n = 5).

Most recently, studies have shown that IL-6 participates in the lineage commitment of pathogenic IL-17-producing T helper cells (T_H_17 cells) from naïve T cells [67]–[69]. On the other hand, IL-17 induces the secretion of IL-6 in a variety of cells including fibroblasts [44], [50] and epithelial cells [51]. We then explored the interaction between IL-6 and IL-17 and determine whether the IL-17/IL-6 axis contributes to the regulation of the keloid niche. Our results showed that incubation with IL-17 enhanced IL-6 expression in both SKPs and KPCs (Figure 5A). However, exposure of KPCs to exogenous IL-6 failed to stimulate IL-17 secretion (data not shown). Similar to IL-6, IL-17 enhanced hTERT and Oct-4 expression in both SKPs and KPCs (Figure 5B). Treatment with IL-6 neutralizing antibody decreased the basal level of hTERT and Oct-4, and in conjunction with IL-17 antibody only slightly augmented the blockade of hTERT and Oct-4 expression in both SKPs and KPCs (Figure 5C). To further delineate the relationship of IL-6 and IL-17 in the regulation of hTERT and Oct-4 expression in SKPs and KPCs, we pretreated cells with neutralizing antibody for either IL-17, or IL-6, or both followed by IL-17 exposure. Our results indicated that IL-17-induced hTERT and Oct-4 expression was abolished by pretreatment with neutralizing antibody for either IL-17, or IL-6, or both (Figure 5D). However, IL-6-induced up-regulation of hTERT and Oct-4 expression was only negated by pre-treatment with IL-6 neutralizing antibody, not by IL-17 neutralizing antibody (Figure 5E). Meanwhile, we examined whether treatment with IL-6 and IL-17 neutralizing antibodies had any effects on cell proliferation and cell viability of SKPs and KPCs. Our results showed that treatment with IL-6, but not IL-17, neutralizing antibodies moderately inhibited BrdU incorporation (*P*<0.05) (Figure S5C), but had no obvious effects on cell viability (Figure S5B). We observed no apparent morphologic change characteristic of apoptosis after treatment with IL-6 and/or IL-17 neutralizing antibodies (data not shown). In all experiments, an isotype-matched antibody (mouse IgG) was used as negative controls. Taken together, these results suggest that IL-17 induces hTERT and Oct-4 expression in SKPs and KPCs by orchestrating the downstream IL-6 paracrine loop.

### IL-17/IL-6 axis contributes to the fibroproliferative growth of KPCs-generated transplants

**Figure 6:**
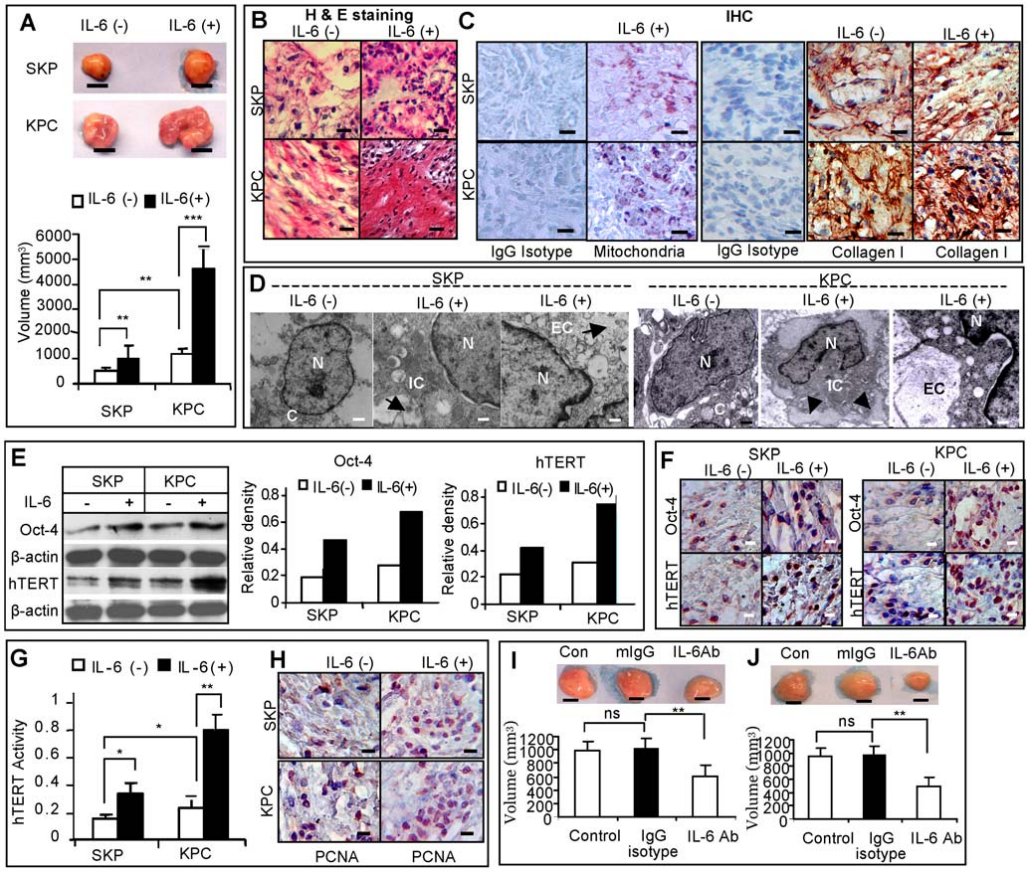
*In vivo* effect of IL-6-linked hydrogel on growth of transplanted KPCs and SKPs. *A*, KPCs or SKPs (2×10^6^) were mixed with hydrogel incorporated with or without IL-6 and subcutaneously injected into immunocompromised mice for 8 weeks. Size and volume of transplants generated from KPCs and SKPs were measured (mean±SEM). Scale bars, 1 mm. ** *P*<0.01; *** *P*<0.001. *B*, Histological analysis of KPC and SKP transplants by hematoxylin and eosin (H & E) staining. Scale bars, 50 µm. *C*, Immunohistochemical studies (IHC) of transplants of KPCs and SKPs using antibody specific for human mitochondria (purple color) and type I collagen (brown color) or an isotype-matched control IgG (IgG isotype), and then counterstained with hematoxylin (nuclei, blue). Scale bars, 50 µm. *D*, Increased synthesis and secretion of collagen by IL-6 in KPCs *versus* SKPs generated transplants as demonstrated by electron microscopy (EM). N, nucleus; C, cytoplasm; IC, intracellular collagen; EC, extracellular collagen. Scale bars, 500 nm. *E*, Expression of Oct-4 and hTERT in KPC and SKP transplants with or without IL-6 as determined by Western blot analysis. *F*, Expression of Oct-4 and hTERT in KPC and SKP transplants with or without IL-6 as determined by immunohistochemical studies. Scale bars, 50 µm. *G*, Telomerase enzyme activity of SKPs and KPCs transplants with or without IL-6 as determined by Telo*TAGGG* Telomerase PCR ELISA (mean±SEM). * *P*<0.05; ** *P*<0.01. *H*, Increased expression of proliferating cell nuclear antigen (PCNA) in transplants of SKPs and KPCs in the presence of IL-6 as determined by immunohistochemical studies. Scale bars, 50 µm. *I* and *J*, Treatment with neutralizing antibodies against IL-6 inhibited *in vivo* formation and growth maintenance of KPC transplants. KPCs were mixed with hydrogel with or without IL-6 neutralizing antibody (IL-6Ab, 10 µg/ml) and injected subcutaneously into immunocompromised mice. KPC transplants were evaluated after 8 weeks (I). To test the inhibitory effect of IL-6 neutralizing antibody on growth of KPC-derived transplant, IL-6 neutralizing antibody was locally injected into KPC-transplants twice a week (5 µg/time) for another 4 weeks (J). An isotype-matched normal mice IgG (IgG isotype) was used as negative controls. The results are representative of five independent experiments using KPCs and the matched SKPs from different patient donors (mean±SEM). ** *P*<0.01; ns, no significance.

In order to recapitulate the distinct “pathological” niche of keloid, we engineered the *in vitro* inflammatory niche by incorporating IL-6 non-covalently into the hydrogel carrier. KPCs and SKPs (2×10^6^) were mixed with IL-6-linked carrier and immediately injected subcutaneously on the dorsal surface of immunocompromised mice. Our results showed that KPCs-generated transplant grew faster than that from SKPs, and the presence of IL-6 in hydrogel carrier significantly promoted the *in vivo* growth of both KPCs and SKPs (Figure 6A). Histological analyses revealed significant increase in cellular components and type I collagen expression in all transplants using IL-6-retained hydrogel as compared to hydrogel only (Figures 6B and 6C). The collagen structure of IL-6 treated transplant was more fibrillar and less amorphous. Importantly, abundant keloid-like thick collagen fibrils were observed in KPC transplants treated with IL-6 as demonstrated by histological and immunohistological staining (Figures 6B and 6C), and electron microscopy (Figure 6D). The unique pattern of excessive intracellular and extracellular accumulation of collagen seen in KPC transplants resembled the abundant collagen matrix observed in keloid tissues (Figure S1B). Meanwhile, we demonstrated that transplants generated from KPCs and SKPs in the presence of IL-6 expressed higher levels of Oct-4 and hTERT, as well as an elevated hTERT enzyme activity, as compared to control (Figures 6E, 6F and 6G). The increased proliferation of dermal stem cells by IL-6 was further confirmed by immunohistochemical staining with antibody for human PCNA (proliferating cell nuclear antigen) (Figure 6H). These results provide further evidence that IL-6, as a downstream target of IL-17 and an essential component of the unique inflammatory niche of keloid, plays a critical role in the regulation of self-renewal and differentiation of keloid stem cells.

To explore whether IL-6 played a role in the initiation of KPC-generated transplants, KPCs mixed with hydrogel with or without human IL-6 neutralizing antibody were injected subcutaneously into immunocompromised mice whereas isotype-matched antibody (mouse IgG) was used as negative control. Our results indicated that treatment with IL-6 neutralizing antibody significantly suppressed the *in vivo* growth of KPC-generated transplants (Figure 6I). We next examined whether IL-6 was capable of maintaining the growth of KPC-generated transplants. KPCs mixed with hydrogel were injected subcutaneously into immunocompromised mice. Four weeks later, IL-6 neutralizing antibody was injected locally into the transplant, twice a week (5 µg/injection) for 4 weeks. Our results showed that intra-lesional injection with IL-6 neutralizing antibody significantly reduced the size of KPC-generated transplants (Figure 6J). These results provide further evidence that IL-6 plays an important role in both initiation and maintenance of KPC transplants, suggesting a potential anti-scarring therapeutic effect.

**Figure 7:**
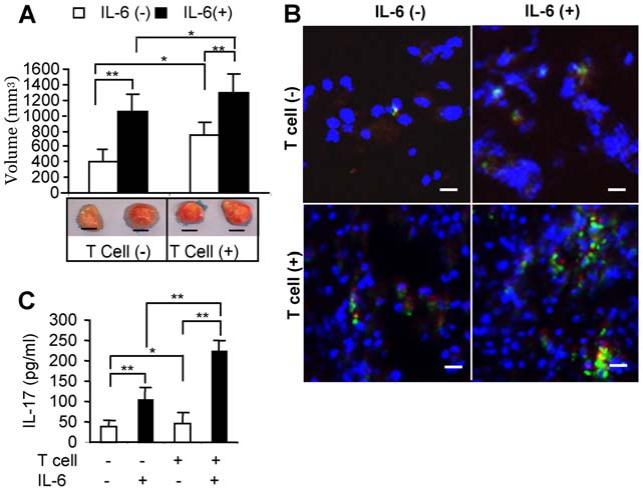
Effect of T cell infusion on i*n vivo* growth of KPC-generated transplants. *A*, Size and volume of KPC transplants (mean±SEM). Activated CD4^+^CD25^−^ T lymphocytes were infused at 1×10^6^/mouse through the tail vein two days before KPC transplants. KPCs were mixed with hydrogel with or without IL-6 and transplanted subcutaneously for 8 weeks. * *P*<0.05; ** *P*<0.01. *B*, Dual-color immunostaining for mouse CD4 (red) and IL-17 (green) using paraffin-embedded sections of KPC transplants. Scale bars, 50 µm. (**C**) Tissue lysates were prepared from the transplant tissues, and IL-17 expression was analyzed by ELISA (mean±SEM). * *P*<0.05, ** *P*<0.01. The results are representative of five independent experiments using KPCs and the matched SKPs from different patient donors.

Since our KPC transplant studies were carried out in athymic nude (nu/nu) mouse which might be devoid of functional T lymphocytes [70], we transplanted KPCs using hydrogel with and without IL-6 into immunocompromised mice adapted by CD4^+^CD25- T-lymphocytes (Naïve T) infusion. Our results showed that boosting T cell function in immunocompromised mice significantly promoted growth of KPC transplants, in the presence or absence of IL-6 (Figure 7A). Immunofluorescence studies showed an increased expression of IL-17 by CD4 positive-T-lymphocytes, which was further enhanced by IL-6 treatment (Figure 7B). The increased IL-17 expression was further confirmed by ELISA assay (Figure 7C). All together, our results suggest that the IL-17/IL-6 axis may function to maintain the highly elevated IL-6 level in the distinct keloid niche with abilities to regulate key stem cell functions such as hTERT and Oct-4 expression, thus contributing to the formation of the benign keloid growth.

## Discussion

Unlike a more precise topographical organization of epithelial stem cells in skin epithelium, the dermal layer harbors Oct-4 and SSEA-4 positive cells that are randomly distributed in the reticular dermis, interspersed among thick ECM, as seen abundantly resided in keloid tissues. The relatively abundant expression of two major markers for embryonic and mesenchymal stem cells [54], [64], [65], Oct-4 and SSEA-4, in keloid as compared to normal skin tissues, suggests the presence of postnatal stem cells in pathologic scar and may contribute to its persistent growth.

In the last few decades, several groups have shown that multipotent precursor cells can be generated from different normal adult human tissues [64]–[66], [71], [72], and may share common biological properties, including the expression profile of various cell surface markers and embryonic transcription markers as well as their multipotent differentiation capabilities [10], [66], [71]. Thus, adult stem cells may play an essential role in growth, homeostasis and regeneration of many tissues. However, up to date, how stem cells contribute to pathologic diseases remains largely unknown. Recently, several studies have reported the isolation and characterization of precursor cells from normal rodent and human normal skin dermis (SKPs) [54]–[56], and recently, mesenchymal-like stem cells from keloid scars (KMLSCs) [73]. However, to our knowledge, no study has determined whether SKPs differ from precursor cells derived from their aberrant scars, specifically their *in vivo* self-renewal and regenerative capacities, and more importantly, whether the pathological niche driven by the inflammatory cytokines of the keloid scar affect their growth and differentiation. In this study, we have isolated and characterized a population of keloid derived precursor cells, termed KPCs, which share some stem cell properties as KMLSCs [73], including self-renewal, clonogenecity and multiple differentiation capacities. However, KPCs inherently expressed higher levels of pluripotent-specific transcription factors Oct-4 and telomerase activity, increased clonogenicity and proliferative capabilities as compared to SKPs derived from the same donor. Based on these distinct stem cell properties, KPCs may represent the benign tumor-like stem cell counterpart of SKPs which might contribute, at least in part, to the high proliferative state in keloid tumor.

More importantly, accumulating evidence has pointed to the critical role of stem cell niche in the regulation of stem cell functions. The balance between proliferation-inhibiting and proliferation-promoting signals provided by the specialized niche microenvironment is the key to homeostatic regulation of stem cell maintenance versus tissue regeneration [12]. Therefore, any changes in the niche components will lead to alterations of stem cell functions. A decline in signaling from the niche that is essential to the maintenance of stem cells can lead to aging and even loss of stem cell number and function [74], [75], thus contributing to aging or degenerative diseases. On the other hand, deregulation or alteration of the niche by dominant proliferation-promoting signals may lead to overgrowth of resident stem cells, thus contributing to hyper-proliferative diseases, benign and malignant tumors [12]. Keloid, a fibro-proliferative benign tumor of skin, is characterized by chronic inflammation, an increased infiltration of inflammatory cells, an enriched milieu of cytokines and growth factors, and an abundant accumulation of ECM [1], [2], [57], thus providing a unique inflammatory niche for the resident stem cells. Using keloid as a niche-related disease model we explore the critical niche components and their role in the regulation of stem cell functions.

IL-6, an inflammatory cytokine, plays a critical role in the pathogenesis of most fibrotic diseases [18] and chronic inflammation-associated tumorigenesis [23], [24]. Consistent with previous reports [5], [6], we have demonstrated that IL-6 is significantly up-regulated in keloid scars versus their normal skin counterparts, and KPCs constitutively express a higher level of IL-6 and IL-6R compared to SKPs. Moreover, our results show that IL-6 can significantly stimulate the expression of both Oct-4 and telomerase, two important stem cell function genes, in cultured KPCs and SKPs, and incubation with specific IL-6 neutralizing antibodies suppressed the constitutive expression of telomerase in KPCs. These findings support our hypothesis that the locally enriched IL-6 in keloid scars might constitute a major component of the inflammatory niche where KPCs reside and play a critical role in the regulation of KPC functions.

Since an animal model for keloid scar does not currently exist, we proposed to generate keloid tumor using dermal derived precursor cells. To recapitulate the “pathological” niche of keloid, we utilized a newly introduced carrier Extracel-HP^TM^ Hydrogel conjugated with IL-6 for the *in vivo* transplantation of KPCs [61], [62]. We have demonstrated that IL-6 stimulates the expression of Oct-4 and telomerase *in vivo* and significantly promotes the growth of KPCs and SKPs-generated transplants. More importantly, abundant keloid-like collagen fibrils were accumulated in transplants of KPCs in the presence of IL-6 that resembled the characteristic histological features of keloid scars. Using the current KPCs-generated transplant as a keloid model we attempted to treat with IL-6 neutralizing antibody using standard intra-lesional injection approach and showed significant reduction in keloid size. Furthermore, pre-treatment of KPCs with IL-6 neutralizing antibody significantly suppressed the *in vivo* growth of KPCs-generated transplant, suggesting a potential anti-scarring preventive and therapeutic effect. Taken together, these important findings further support the notion that IL-6, as a major component of the inflammatory niche in keloid scars, may play a critical role in the acquirement of benign tumor-like stem cell phenotypes, therefore the benign tumor growth.

IL-17, another recently identified inflammatory cytokine, exclusively produced by activated T cells and whose receptor is widely expressed in many cell types [42], plays a pivotal role in several inflammatory diseases [38]–[41]. IL-6, in concert with TGF-β, induces T_H_17 cell differentiation from naïve T cells by orchestrating a series of “downstream” cytokine-dependent signaling pathways [67]–[69]. More importantly, IL-6 has been reported to function both upstream and downstream of IL-17, constituting a positive feedback loop that promotes autoimmune and allergic diseases [50], [51]. In this study, we have demonstrated for the first time to our knowledge that IL-17 is up-regulated in keloid tissues, and KPCs express relatively higher IL-17R than normal skin-derived precursor cells. Furthermore, we demonstrated that blocking IL-6 abolished IL-17-induced up-regulation of hTERT and Oct-4 expression; however, blocking IL-17 failed to attenuate IL-6-induced up-regulation of hTERT and Oct-4 expression in KPCs. These findings support our hypothesis that elevated IL-6 and TGF-β in keloids promote differentiation of T_H_17 cells, which in turn secretes more IL-17 to further enhance the release of IL-6 from keloid stem cells or other stromal cells. The IL-17-triggered positive-feedback loop *via* paracrine IL-6 induction constitutes the “pathological” niche or altered inflammatory niche that drives KPCs toward proliferation, and thus tumor-like keloid growth (Figure S6). However, it is important to emphasize that despite the tumor-like growth behavior, KPCs were distinct from cancer cells or cancer stem-like cells (25) in that KPCs displayed a normal karyotype and an absence of cytogenetic abnormalities. Since inflammation plays a critical role in tumorigenesis (23, 24), longer *in vivo* experimental observation, up to 2 months in our study, was carried out to determine the long-term effects of inflammatory niche components, specifically the IL-17/IL-6 axis, on the acquired properties of KPCs, including their replicative/proliferative capabilities, and cellular transformation. Due to the limitation of the biological activity of the hydrogel-released growth factors, we were not able to prolong the study beyond the above time point. Further studies will be needed to determine the effect of chronic inflammation on potential tumorigenesis.

In addition to providing proliferation and differentiation signals, stem cell niche also provides homing signals to recruit bone marrow MSCs to sites of injury and inflammation [10], [12], whereby a variety of inflammatory mediators, including hypoxia, reactive oxygen species (ROS), inflammatory chemokines and cytokines, may trigger the migration of BMMSCs [76]. In response to the unique tumor microenvironment of chronic inflammation [77], [78], BMMSCs may acquire the cancer-associated fibroblast (CAF) phenotype and subsequently promote tumor growth and metastasis *via* the production of pro-angiogenic and tumor stimulating paracrine factors [79], [80]. Using a dual-chamber cell-migration assay, keloid-derived fibroblasts have been shown to induce human BMMSCs chemoattraction toward keloid cells, and develop myofibroblastic changes characterized by abundant rough endoplasmic reticulum, secretion of collagen-like fibers as well as actin-type microfilament bundles [7]. These findings suggest that the unique keloid microenvironment or niche may preferentially recruit bone marrow MSCs and sustain their interaction with resident fibroblasts, thus contributing to keloid formation by producing abundant extracellular matrix at the scar site. The interaction between the keloid niche and keloid derived stem cells, KPCs, has been demonstrated here in our study, which is sustained by the IL-17/IL-6 feedback loop. It remains to be determined whether IL-17/IL-6 axis contributes to the recruitment of BMMSCs to keloid lesion and whether KPCs represent a distinct population of resident MSCs or derived from BMMSCs. In addition, a recent study reported that methylprednisolone is capable of inhibiting IL-17 production in lymphocytes and lymph node cells [81]. Further studies are needed to determine whether the anti-scarring effect of the intralesional steroid therapy in keloid scar correlates with its role on the regulation of the IL-17/IL-6. Using our established keloid-like animal models we will be able to further investigate the interplay between stem cells and their immediate niche.

In summary, we have successfully identified multipotent precursor cells from keloid scars and provided evidence supporting IL-17/IL-6 axis as an essential component of the unique keloid niche that provides extra proliferation-promoting signals to resident stem cells. Based on our findings, we postulate that the inflammatory stimuli, i.e., inflammatory infiltrates and enriched cytokines, induced by wounding, surgical injury, or infection, may produce a persistent state of chronic inflammation at the wound site. The increased IL-6, a major component of the inflammatory niche, in conjunction with TGFβ1, may trigger the differentiation of naïve T cells into T_H_17 cells, which further stimulate IL-6 secretion via IL-17, thus creating an enriched pro-inflammatory cytokine milieu. The functionally altered niche can promote the resident stem cells to acquire a benign tumor-like stem cell phenotype characterized by increased cell proliferation and differentiation, therefore directly drives the benign tumor growth as seen in keloids (Figure S6). Findings from this study have not only substantiated our current knowledge regarding the potential role of adult stem cells in pathologic diseases, specifically skin fibrosis, and other fibro-proliferative disorders, but most importantly, have provided a promising niche-related disease model, to further explore the intricate interactions between stem cells and their functional niche components, and ultimately lead to the development of an animal model for keloid fibrosis. Finally, this study should open a new avenue for stem cell research on benign tumor and lead to the rational design and development of innovative methods for prevention and treatment of niche-related diseases by specifically modulating or targeting the unique niche microenvironment of adult stem cells.

## Supporting Information

Methods S1 (0.05 MB DOC)

Table S1 Antibodies for immunostaining and neutralization
(0.09 MB PDF)

Table S2 RT-PCR primer sequences
(0.06 MB PDF)

Table S3 Quantification of cytokine antibody array
(0.05 MB PDF)

Table S4 Transplantation with a range of number of dermal stem cells
(0.01 MB PDF)

Figure S1 Isolation of precursor cells from keloid tissues. (A) Keloid scar displays benign tumor phenotype in terms of growth and recurrence. (B) H&E histological stain of keloid tissues and the matched peripheral normal skins. PD: papillary dermis; RD: reticular dermis. Scale bars, 50 µm. (C) Semi-quantification of immunohistochemical staining of Oct-4 and SSEA-4 in keloid and the matched normal skin (Fig. 1A) as described in Materials & Methods. **P<0.01. (D) Colony formation of stem cells derived from keloid (KPCs) and normal skin (SKPs). (E) Subcloning and culture of mesenchymal stem cells from keloids (KPCs) in α-MEM medium supplemented with 10% FBS, 1 x NEAA (non-essential amino acid) and antibiotics. Scale bars, 100 µm. (F) Determination of doubling time of KPCs and SKPs as described in Materials and Methods (mean±SEM). (G) Karyotyping of the SKP and KPC clones at passage 10. The results are representative of at least five independent experiments.
(7.14 MB TIF)

Figure S2 Multipotent differentiation of keloid derived precursor cells (KPCs). (A–C) Adipogenic differentiation of SKP or KPC as determined by Oil Red O staining (A and B) and RT-PCR analysis of specific adipocyte genes (C). (D–F) Osteogenic differentiation as determined by Alizarin Red S staining (D and E) and RT-PCR analysis of osteocalcin gene (F). Human bone marrow mesenchymal stem cells (hBMMSCs) were used as positive controls whereas KPCs culturing under normal growth medium were served as non-induction control. Scale bars, 50 µm. Data are representative of at least five independent experiments using KPCs and the matched SKPs from 5 different patient donors (mean±SEM). * P<0.05; ns, no significance.
(5.20 MB TIF)

Figure S3 Sphere-colony formation of KPCs. (A) Subcloning and expansion of sphere-colonies derived from keloids in DMEM-LG/F12 (3∶1) supplemented with 40 ng/mL FGF-2, 20 ng/mL EGF, B27 and antibiotics. Scale bars, 100 µm. (B and C) Expression of stem cell markers and BrdU incorporation by keloid-derived sphere colonies (K1∼K3) as determined by immunofluorescence staining (B) and RT-PCR analysis (C). Scale bars, 50 µm. (D) Multipotent differentiation of keloid-derived sphere colonies into different lineages of neural cells, adipocytes, and osteocytes as determined by immunofluorescence staining with specific neural cell markers, Oil Red O and von Kossa staining. Scale bars, 50 µm. The results are representative of at lease five independent experiments.
(4.37 MB TIF)

Figure S4 Expression of IL-6 receptor (IL-6R) in keloids (K1, K2) and matched normal skins (N1, N2). (A) Paraffin-embedded sections of keloid and the matched normal skin were immunostained with a specific antibody for human IL-6R or an isotype-matched control IgG. Scale bars, 50 µm. (B) Western blot analysis of IL-6R in tissue lysates. (C) Flow cytometric analysis of IL-6R and IL-17R expression in cultured SKPs or KPCs. The results are representative of five independent experiments.
(1.72 MB TIF)

Figure S5 Effect of IL-6 on the expression of SSEA-4 and BrdU incorporation in SKPs and KPCs. (A) Cells were stimulated with 20 ng/ml IL-6 for 24 h followed by immunostained with antibodies for SSEA-4 and FITC-conjugated secondary antibody and analyzed by flow cytometry. (B and C) Effects of IL-6 and IL-17 neutralizing antibodies on cell viability and proliferation in SKPs and KPCs. Cells were treated for 24 h with 5 µg/ml of neutralizing antibodies for human IL-6, or IL-17, or both in the absence of IL-6 and IL-17, whereby an isotype-matched normal mice antibody (mIgG) was used as negative controls. Cell viability and proliferation were determined by MTT (B) and BrdU incorporation assay (C), respectively. The results are representative of at least five independent experiments (mean±SEM). #, no significant difference; * P<0.05, as compared with non-treatment control.
(0.45 MB TIF)

Figure S6 Schemed inflammatory niche-driven benign tumor growth model. Under the chronic inflammatory microenvironment, keloid-derived precursor cells (KPCs) are persistently interacted with inflammatory cells and stimulated by enriched milieu of pro-inflammatory mediators, specifically IL-6, and then acquired a benign tumor-like stem cell phenotype characterized by moderately increased telomerase activity, and consequently, an increased proliferative capacity, whereby the increased IL-17 continuously drives this process by augmenting the production of IL-6 by KPCs, thus leading to the overgrowth of keloid benign tumor.
(0.87 MB TIF)

## References

[1] Niessen FB, Spauwen PHM, Schalkwijk J, Moshe K (1999). On the nature of hypertrophic scars and keloids: a review.. Plast Reconstr Surg.

[2] Kelly AP (2004). Medical and surgical therapies for keloids.. Dermatol Ther.

[3] Ketchum LD, Robinson DW, Masters FW (1971). Follow-up on treatment of hypertrophic scars and keloids with triamcinolone.. Plast Reconstr Surg.

[4] Lee JY, Yang CC, Chao SC, Wong TW (2004). Histopathological differential diagnosis of keloid and hypertrophic scar.. Am J Dermatopathol.

[5] Xue H, McCauley RL, Zhang W (2000). Elevated interleukin-6 expression in keloid fibroblasts. J Surg. Res.

[6] Ghazizadeh M, Tosa M, Shimizu H, Hyakusoku H, Kawanami O (2007). Functional implications of the IL-6 signaling pathway in keloid pathogenesis.. J Invest Dermatol.

[7] Akino K, Akita S, Yakabe A, Mineda T, Hayashi T (2008). Human mesenchymal stem cells may be involved in keloid pathogenesis.. Int J Dermatol.

[8] Li L, Xie T (2005). Stem cell niche: structure and function.. Annu Rev Cell Dev Biol.

[9] Schofield R (1978). The relationship between the spleen colony-forming cell and the haemopoietic stem cell.. Blood Cells.

[10] Kolf CM, Cho E, Tuan RS (2007). Biology of adult mesenchymal stem cells: regulation of niche, self-renewal and differentiation.. Arthritis Res Ther.

[11] Scadden DT (2006). The stem-cell niche as an entity of action.. Nature.

[12] Li LH, Neaves WB (2006). Normal stem cells and cancer stem cells: the niche matters.. Cancer Res.

[13] Moore KA, Lemischka IR (2006). Stem cells and their niches.. Science.

[14] Xie T, Li LH (2007). Stem cells and their niche: an inseparable relationship.. Development.

[15] Gauldie J, Richards C, Harnish D, Lansdorp P, Baumann H (1987). Interferon β2/B-cell stimulatory factor type 2 shares identity with monocyte-derived hepatocyte-stimulating factor and regulates the major acute phase protein response in liver cells.. Proc Natl Acad Sci USA.

[16] Kopf M, Baumann H, Freer G, Freudenberg M, Lamers M (1994). Impaired immune and acute-phase responses in interleukin-6-deficient mice.. Nature.

[17] Kaplanski G, Marin V, Montero-Julian F, Mantovani A, Farnarier C (2003). IL-6: a regulator of the transition from neutrophil to monocyte recruitment during inflammation.. Trends Immunol.

[18] Gabay C (2006). Interleukin-6 and chronic inflammation.. Arthritis Res Ther.

[19] Tan P, Farmiloe S, Yeoman S, Watson J (1990). Expresssion of IL-6 gene in rheumatoid synovial fibroblasts.. J Rheumatol.

[20] Feghali CA, Bost KL, Bouware DW, Levy LS (1994). Control of IL-6 expression and response in fibroblasts from patients with systemic sclerosis.. Autoimmunity.

[21] Xu G, Zhang Y, Zhang L, Ren G, Shi Y (2007). The role of IL-6 in inhibition of lymphocyte apoptosis by mesenchymal stem cells.. Biochem Biophys Res Commun.

[22] Kovacević-Filipović M, Petakov M, Hermitte F, Debeissat C, Krstić A (2007). Interleukin-6 (IL-6) and low O_2_ concentration (1%) synergize to improve the maintenance of hematopoietic stem cells (Pre-CFC).. J Cell Physiol.

[23] Bollrath J, Phesse TJ, von Burstin VA, Putoczki T, Bennecke M (2009). gp130-mediated stat-3 activation in enterocytes regulates cell survival and cell-cycle progression during colitis-associated tumorigenesis.. Cancer Cell.

[24] Grivennikov S, Karin E, Terzic J, Mucida D, Yu GY (2009). Il-6 and Stat-3 are required for survival of intestinal epithelial cells and development of colitis-associated cancer.. Cancer Cell.

[25] Sansone P, Storci G, Tavolari S, Guarnieri T, Giovannini C (2007). IL-6 triggers malignant features in mammospheres from human ductal breast carcinoma and normal mammary gland.. J Clin Invest.

[26] Akiyama M, Hideshima T, Hayashi T, Tai YT, Mitsiades CS (2002). Cytokines modulate telomerase activity in a human multiple myeloma cell line.. Cancer Res.

[27] Yamagiwa Y, Meng FY, Patel T (2006). Interleutin-6 decreases senescence and increases telomerase activity in malignant human cholangiocytes.. Life Sci.

[28] Lee HW, Blasco MA, Gottlieb GJ, Horner JW, Greider CW (1998). Essential role of mouse telomerase in highly proliferative organs.. Nature.

[29] Rudolph KL, Chang S, Lee HW, Blasco M, Gottlieb GJ (1999). Longevity, stress response, and cancer in aging telomerase-deficient mice.. Cell.

[30] Flores I, Cayuela ML, Blasco MA (2005). Effects of telomerase and telomere length on epidermal stem cell behavior.. Science.

[31] Sarin KY, Cheung P, Gilison D, Lee E, Tennen RI (2005). Conditional telomerase induction causes proliferation of hair follicle stem cells.. Nature.

[32] Harrinton LE, Hatton RD, Mangan PR, Turner H, Murphy TL (2005). Interleukin-17-producing CD4^+^ effector T cells develop via a lineage distinct from the T helper type 1 and 2 lineages.. Nat Immunol.

[33] Bettelli E, Carrier Y, Gao W, Korn T, Strom TB (2006). Reciprocal developmental pathways for the generation of pathogenic effector Th17 and regulatory T cells.. Nature.

[34] Manel N, Unutmaz D, Littman DR (2008). The differentiation of human T (H)-17 cells requires transforming growth factor-beta and induction of the nuclear receptor RORgammat.. Nat Immunol.

[35] Bettelli E, Korn T, Oukka M, Kuchroo VK (2008). Induction and effector functions of TH17 cells.. Nature.

[36] Yang L, Anderson DE, Baecher-Allan C, Hastings WD, Bettelli E (2008). IL-21 and TGF-beta are required for differentiation of human T (H)17 cells.. Nature.

[37] Volpe E, Servant N, Zollinger R, Bogiatzi SI, Hupé P (2008). A critical function for transforming growth factor-beta, interleukin 23 and proinflammatory cytokines in driving and modulating human T (H)-17 responses.. Nat Immunol.

[38] Nakae S, Saijo S, Horai R, Sudo K, Mori S (2003). IL-17 production from activated T cells is required for the spontaneous development of destructive arthritis in mice deficient in IL-1 receptor antagonist.. Proc Natl Acad Sci USA.

[39] Murphy CA, Langrish CL, Chen Y, Blumenschein W, McClanahan T (2003). Divergent pro- and anti-inflammatory roles for IL-23 and IL-12 in joint autoimmune inflammation.. J Exp Med.

[40] Lubberts E, Koenders MI, van den Berg WB (2005). The role of T-cell interleukin-17 in conducting destructive arthritis: lessons from animal models.. Arthritis Res Ther.

[41] Fujino S, Andoh A, Bamba S, Ogawa A, Hata K (2003). Increased expression of interleukin 17 in inflammatory bowel disease.. Gut.

[42] Yao Z, Fanslow WC, Seldin MF, Rousseau AM, Painter SL (1995). Herpesvirus Saimiri encodes a new cytokine, IL-17, which binds to a novel cytokine receptor.. J Immunol.

[43] Aggarwal S, Gurney AL (2002). IL-17: prototype member of an emerging cytokine family.. J Leukoc Biol.

[44] Hwang S-Y, Kim JY, Kim KW, Park MK, Moon Y (2004). IL-17 induces production of IL-6 and IL-8 in rheumatoid arthritis synovial fibroblasts via NKκB- and PI3-kinase/Akt-dependent pathways.. Arthritis Res Ther.

[45] Ruddy MJ, Wong GC, Liu XK, Yamamoto H, Kasayama S (2004). Functional cooperation between interleukin-17 and tumor necrosis factor-α is mediated by CCAAT/enhancer-binding protein family members.. J Biol Chem.

[46] Huang F, Kao C-Y, Wachi S, Thai P, Ryu J (2007). Requirement for both JAK-mediated PI3K signaling and ACT1/TRAF6/TAK1-dependent NF-κB activation by IL-17A in enhancing cytokine expression in human airway epithelial cells.. J Immunol.

[47] Patel DN, King CA, Bailey SR, Holt JW, Venkatachalam K (2007). Interleukin-17 stimulates C-reactive protein expression in hepatocytes and smooth muscle cells via p38 MAPK and ERK1/2-dependent NKκB and C/EBP β activation.. J Biol Chem.

[48] Jovanovic DV, Di Battista JA, Martel-Pelletier J, Jolicoeur FC, He Y (1998). IL-17 stimulates the production and expression of proinflammatory cytokines, IL-beta and TNF-alpha, by human macrophages.. J Immunol.

[49] Shalom-Barak T, Quach J, Lotz M (1998). Interleukin-17-induced gene expression in articular chondrocytes is associated with activation of mitogen-activated protein kinases and NF-kappaB.. J Biol Chem.

[50] Ogura H, Murakami M, Okuyama Y, Tsuruoka M, Kitabayashi C (2008). Interleukin-17 promotes autoimmunity by triggering a positive-feedback loop via interleukin-6 induction.. Immunity.

[51] Chen Y, Thai P, Zhao YH, Ho YS, DeSouza MM (2003). Stimulation of airway mucin gene expression by interleukin (IL)-17 through IL-6 paracrine/autocrine loop.. J Biol Chem.

[52] Huang H, Kim HJ, Chang E-J, Lee ZH, Hwang SJ (2009). IL-17 stimulates the proliferation and differentiation of human mesenchymal stem cells: implications for bone remodeling..

[53] Bartsch G, Yoo JJ, De Coppi P, Siddiqui MM, Schuch G (2005). Propagation, expansion, and multilineage differentiation of human somatic stem cells from dermal progenitors.. Stem Cell Develop.

[54] Toma JG, Akhavan M, Fernandes KJ, Barnabé-Heider F, Sadikot A (2001). Isolation of multipotent adult stem cells from the dermis of mammalian skin.. Nat Cell Biol.

[55] Fernandes KJ, McKenzie IA, Mill P, Smith KM, Akhavan M (2004). A dermal niche for multipotent adult skin-derived precursor cells.. Nat Cell Biol.

[56] Toma JG, McKenzie IA, Bagli D, Miller FD (2005). Isolation and characterization of multipotent skin-derived precursors from human skin.. Stem Cells.

[57] Zhang QZ, Wu Y, Ann DK, Messadi DV, Tuan TL (2003). Mechanisms of hypoxic regulation of plasminogen activator inhibitor-1 gene expression in keloid fibroblasts.. J Invest Dermatol.

[58] Bi YM, Ehirchiou D, Kilts TM, Inkson CA, Embree MC (2007). Identification of tendon stem/progenitor cells and the role of the extracellular matrix in their niche.. Nat Med.

[59] Shi S, Gronthos S, Chen S, Reddi A, Counter CM (2002). Bone formation by human postnatal bone marrow stromal stem cells is enhanced by telomerase expression.. Nat Biotech.

[60] You S, Moon JH, Kim TK, Kim SC, Kim JW (2004). Cellular characteristics of primary and immortal canine embryonic fibroblast cells.. Exp Mol Med.

[61] Cai S, Liu Y, Shu XZ, Prestwich GD (2005). Injectable glycosaminoglycan hydrogels for controlled release of human basic fibroblast growth factor.. Biomaterials.

[62] Pike DB, Cai S, Pomraning KR, Firpo MA, Fisher RJ (2006). Heparin-regulated release of growth factors *in vitro* and angiogenic response *in vivo* to implanted hyaluronan hydrogels containing VEGF and bFGF.. Biomaterials.

[63] Yamaza T, Miura Y, Bi Y, Liu Y, Akiyama K (2008). Pharmacological stem cell based intervention as a new approach to osteoporosis treatment in rodents.. PLoS One.

[64] De Coppi P, Pozzobon M, Piccoli M, Gazzola MV, Boldrin L (2006). Isolation of mesenchymal stem cells from human vermiform appendix.. J Surg Res.

[65] Gang EJ, Bosnakovski D, Figueiredo CA, Visser JW, Perlingeiro CR (2007). SSEA-4 identifies mesenchymal stem cells from bone marrow.. Blood.

[66] Beltrami AP, Cesselli D, Bergamin N, Marcon P, Rigo S (2007). Multipotent cells can be generated in vitro from several adult human organs (heart, liver, and bone marrow).. Blood.

[67] Kimura A, Naka T, Kishimoto T (2007). IL-6-dependent and –independent pathways in the development of interleukin 10-producing T helper cells.. Proc Natl Acad Sci USA.

[68] McGeachy MJ, Bak-Jensen KS, Chen Y, Tato CM, Blumenschein W (2007). TGF-β and IL-6 drive the production of IL-17 and IL-10 by T cells and restrain T_H_-17 cell-mediated pathology.. Nat Immunol.

[69] Zhou L, Ivanov II, Spolski R, Min R, Shenderov K (2007). IL-6 programs T_H_-17 cell differentiation by promoting sequential engagement of the IL-21 and IL-23 pathways.. Nat Immunol.

[70] MacDonald HR (1984). Phenotypic and functional characteristics of ‘T-like’ cells in nude mice.. Exp Cell Biol.

[71] Izadpanah R, Trygg C, Patel B, Kriedt C, Dufour J (2006). Biologic properties of mesenchymal stem cells derived from bone marrow and adipose tissue.. J Cell Biochem.

[72] da Silva ML, Chagastelles PC, Nardi NB (2006). Mesenchymal stem cells reside in virtually all post-natal organs and tissues.. J Cell Sci.

[73] Moon J-H, Kwak SS, Park G, Jung H-Y, Yoon BS (2008). Isolation and characterization of multipotent human keloid-derived mesenchymal-loke stem cells.. Stem Cell Dev.

[74] Boyle M, Wong C, Rocha M, Jones DL (2007). Decline in self-renewal factors contributes to aging of the stem cell niche in the *Drosophila* testis.. Cell Stem Cell.

[75] Pan L, Chen S, Weng C, Call G, Zhu D (2007). Stem cell aging is controlled both intrinsically and extrinsically in the *Drosophila* ovary.. Cell Stem Cell.

[76] Spaeth E, Klopp A, Dembinski J, Andreeff M, Marini F (2008). Inflammation and tumor microenvironments: defining the migratory itinerary of mesenchymal stem cells.. Gene Ther.

[77] Coffelt SB, Marini FC, Watson K, Zwezdaryk KJ, Dembinski JL (2009). The pro-inflammatory peptide LL-37 promotes ovarian tumor progression through recruitment of multipotent mesenchymal stromal cells.. Proc Natl Acad Sci USA.

[78] Karnoub AE, Dash AB, Vo AP, Sullivan A, Brooks MW (2007). Mesenchymal stem cells within tumour stroma promote breast cancer metastasis.. Nature.

[79] Mishra PJ, Mishra PJ, Humeniuk R, Medina DJ, Alexe G (2008). Carcinoma-associated fibroblast-like differentiation of human mesenchymal stem cells.. Cancer Res.

[80] Spaeth EL, Dembinski JL, Sasser AK, Watson K, Klopp A (2009). Mesenchymal stem cell transition to tumor-associated fibroblasts contributes to fibrovascular network expansion and tumor progression.. PLoS One.

[81] Momcilović M, Miljković Z, Popadić D, Marković M, Savić E (2008). Methylprednisolone inhibits interleukin-17 and interferon-gamma expression by both naive and primed T cells.. BMC Immunol.

