# Supplementary material for: Tumor-Like Stem Cells Derived from Human Keloid Are Governed by the Inflammatory Niche Driven by IL-17/IL-6 Axis
Source: PLoS One. 2009 Nov 11;4(11):e7798. doi: 10.1371/journal.pone.0007798 (PMC2771422; doi:10.1371/journal.pone.0007798)
Supplement: Methods S1 — (0.05 MB DOC) [file pone.0007798.s001.doc]

### Methods S1

**Multipotent differentiation of dermal derived precursor cells**

*Osteogenic differentiation*: SKPs and KPCs were plated at 5 x 10^5^ cells/well in 6-well plate in mesenchymal stem cell (MSC) growth medium, allowed to adhere overnight, and replaced with Osteogenic Induction Medium (PT-3002, Cambrex, Charles City, IA) supplemented with dexamethasone, L-glutamine, ascorbic acid, and β-glycerophosphate. After 4~5 weeks, the i*n vitro* mineralization was assayed by Alizarin red S (Sigma-Aldrich) staining and quantified by acetic acid extraction method (Gregory et al, 2004). Under some conditions, *von* Kossa staining was used to identify extracellular mineralized matrix.

*Adipogenic differentiation*: SKPs and KPCs were plated at 5 x 10^5^ cells/well in 6-well plate in MSC growth medium, allowed to adhere overnight, and replaced with adipogenic induction medium supplemented with 10 μM human insulin, 1 μM dexamethasone, 200 μM indomethacin, and 0.5 mM 3-isobutyl-1-methylxanthine (Sigma-Aldrich, St Louis, MO). Oil Red O staining was performed to detect intracellular lipid vacuoles characteristic of adipocytes, and the dye content was quantified by isopropanol elution (5min shaking) and spectrophotometry at 510 nm (Yu W et al, 2007).

*Neuronal differentiation*: Single cells were dissociated from keloid-derived precursor cell (KPCs) spheres and plated at 5 x 10^3^ cells/well in 8-well chamber slides (Nalge Nunc, Rochester, NY) coated with poly-d-lysine/laminin and cultured in DMEM/F12 (3:1) (Invitrogen, Carlsbad, CA) supplemented with 40ng/ml FGF-2 (Chemicon, Billerica, MA) and 10% FBS (Invitrogen) for 5~7 days. For neurogenic induction cells were cultured for another 5~7 days in the same medium without FGF-2, but with the addition of 10 ng/ml nerve growth factor (NGF), 10 ng/ml brain-derived neurotrophic factor (BDNF), and 10 ng/ml NT-3 (Peprotech, Rocky Hill, NJ). To differentiate KPCs into Schwann cells, dissociated spheres were cultured in DMEM/F12 (3:1) containing 10% FBS for 1 week, then switched to the same medium supplemented with 4μM forskolin (Sigma). In all experiments, cells were induced to differentiate for 2–3 weeks, with 50% of the medium changed every 3–4 days.

**Karyotyping**

Karyotyping was performed by the Cytogenetics Core Laboratory of City of Hope and Beckman Research Institute (<http://www.cityofhope.org/research/support/cytogenetics/Pages/default.aspx>) (Duarte, CA). SKPs and KPCs at passage 10 derived from the same patient were cultured as described in *Materials and Methods* until near confluency. Metaphase spreads were prepared and chromosome analysis was performed according to standard procedures.

**Serial transplantation**

To further confirm the *in vivo* differentiation ability of KPCs and SKPs, we performed serial transplantation as well as transplantation using a limiting dilution assay. For serial transplantation, 2 x 10^6^ KPCs were transplanted subcutaneously into immunocompressed mice. After 4 weeks, the transplants were harvested and recovered single cells were expanded and re-transplanted into immunocompressed mice for another 4 weeks. Meanwhile, various numbers of KPCs and SKPs at 2 x 10^6^, 1 x 10^6^, 5 x 10^5^, 1 x 10^5^, 1 x 10^4^, 1 x 10^3^, and 1 x 10^2^ were transplanted subcutaneously into immunocompromised mice, and 8 weeks later the transplants were harvested.

**Immunohistochemical studies**

The paraffin- or frozen sections were incubated with primary antibodies and detected using the universal immunoperoxidase (HRP) ABC kit (Vector, Burlingame, CA). They were counterstained with hematoxylin. Isotype-matched control antibodies (Invitrogen) were used as negative controls. For histological study, paraffin sections were stained with hematoxylin and eosin (H&E). To quantify Oct-4 and SSEA-4 expression, sections were visualized using a 40X objective, and positively stained cells in 5 random high-power fields (HPF) were counted and expressed as percentage of total cells (Walker, 2006).

**Electron microscopy**

The samples of KPC and SKP transplants were fixed in 2.5% glutaraldehyde and embedded in epoxy resin. The ultra-thin sections (70 μm) were stained with uranyl acetate and lead citrate and observed under a transmission electron microscopy.

##### References

### Gregory CA, Gunn WG, Peister A, and Prockop DJ. An Alizarin red-based assay of mineralization by adherent cells in culture: comparison with cetylpyridinium chloride extraction. *Anal. Biochem.* 2004; 329: 77–84

### Yu W, Chen Z, Zhang J, Zhang L, Ke H, Huang L, Peng Y, Zhang X, Li S, Lahn BT, Xiang AP. Critical role of phosphoinositide 3-kinase cascade in adipogenesis of human mesenchymal stem cells. *Mol. Cell. Biochem.* 2007; 310: **11-18**

Walker RA. Quantification of immunohistochemistry-issues concerning methods, utility and semiquantitative assessment I. *Histopathology* 2006; **49**: 406-410
