# Supplementary material for: Tumor-Like Stem Cells Derived from Human Keloid Are Governed by the Inflammatory Niche Driven by IL-17/IL-6 Axis
Source: PLoS One. 2009 Nov 11;4(11):e7798. doi: 10.1371/journal.pone.0007798 (PMC2771422; doi:10.1371/journal.pone.0007798)
Supplement: Table S1 — Antibodies for immunostaining and neutralization (0.09 MB PDF) [file pone.0007798.s002.pdf]

Table S1: Antibodies for immunostaining and neutralization

Antibodies	Host	Immunoglobulin	Vendor
Actin, beta (β-actin)	*Ms	Monoclonal IgG	Sigma
5-bromo-2-deoxyuridine (BrdU)	Ms	Monoclonal IgG	Sigma
Catenin, beta (β-catenin)	Ms	Monoclonal IgG	Cell signaling
CD3	Ms	Monoclonal IgG	BioLegend
CD29, PE-conjugated	Ms	Monoclonal IgG	BD Bioscience
CD34, PE-conjugated	Ms	Monoclonal IgG	BD Bioscience
CD45, PE-conjugated	Ms	Monoclonal IgG	BD Bioscience
CD73, FITC-conjugated	Ms	Monoclonal IgG	BD Bioscience
CD90, FITC-conjugated	Ms	Monoclonal IgG	BD Bioscience
CD105, FITC-conjugated	Ms	Monoclonal IgG	BD Bioscience
CD146, PE-conjugated	Ms	Monoclonal IgG	BD Bioscience
Mouse CD3ε	Ham	Monoclonal IgG	BD Bioscience
Mouse CD28	Ham	Monoclonal IgG	BD Bioscience
Collagen, type I (Col-I)	Ms	Monoclonal IgG	Sigma
2’, 3’-cyclic nucleotide 3’-phosphodiesterase (CNPase)	Ms	Monoclonal IgG	Sigma
Fibronectin (FN)	Rb	Polyclonal IgG	Sigma
Glial fibrillary acidic protein (GFAP)	Rb	Polyclonal IgG	Sigma
Interleukin 6, for immunostaining (IL-6)	Ms	Monoclonal IgG	Chemicon
Interleukin 6, for neutralization (IL-6Ab)	Ms	Monoclonal IgG	R&D Systems
Interleukin 6 receptor (IL-6R)	Ms	Monoclonal IgG	Chemicon
Interleukin 17, for immunostaining (IL-17)	Go	Polyclonal IgG	Santa Cruz
Interleukin 17, for neutralization (IL-17Ab)	Ms	Monoclonal IgG	R&D Systems
Interleukin 17 receptor (IL-17R)	Ms	Monoclonal IgG	Santa Cruz
Mitochondria, human (Mt)	Ms	Monoclonal IgG	Chemicon
Nestin	Ms	Monoclonal IgG	Sigma
Neurofilament (NFL)	Ms	Monoclonal IgG	Sigma
Octamer-4 (Oct-4)	Rb	Polyclonal IgG	Chemicon
Octamer-4 (Oct-4)	Ms	Monoclonal IgG	Santa Cruz
Proliferative cell nuclear antigen (PCNA)	Rb	Polyclonal IgG	Santa Cruz
Smooth muscle actin, alpha (α-SMA)	Ms	Monoclonal IgG	Sigma
Stage specific embryonic antigen 4 (SSEA-4)	Ms	Monoclonal IgG	Chemicon
Stro-1	Ms	Monoclonal IgM	ATCC
Telomerase reverse transcriptase, human (hTERT)	Ms	Monoclonal IgM	Novus
Tubulin III, beta (β-tubulin III)	Ms	Monoclonal IgG	Sigma
Vitronectin (VN)	Rb	Polyclonal IgG	Sigma

*Go: goat, Ms: mouse, Rb: rabbit, Ham: hamster
