# Supplementary material for: Tumor-Like Stem Cells Derived from Human Keloid Are Governed by the Inflammatory Niche Driven by IL-17/IL-6 Axis
Source: PLoS One. 2009 Nov 11;4(11):e7798. doi: 10.1371/journal.pone.0007798 (PMC2771422; doi:10.1371/journal.pone.0007798)
Supplement: Table S2 — RT-PCR primer sequences (0.06 MB PDF) [file pone.0007798.s003.pdf]

Table S2: RT-PCR primer sequences

Genes	**Upper primer (5’-3’)**	**Lower primer (5’-3’)**
Oct-4	CGCACCACTGGCATTG TCAT	TTCTCCTTGATGTCACGCAC
Rex-1	TGAAAGCCCACA TCCTAACG	CAAGCTATCCTCCTGCTTTGG
Nanog	AATACCTCAGCCTCCAGCAGATG	CTGCGTCACACCATTGCTATTCT
Pax3	CATCCGGCCCTGCGTCATCTC	TGGCCTT CTTCTCGCTTTCCTCTG
Slug	CATCTTTGGGGCGAGTGAGTCC	CCCCCGTGTGAGTTCTAATGTGTC
hTERT	AGCCAGTCTCACCTTCAACCGC	GGAGTAGCAGAGGGAGGCCG
LPL	CTGGTCGAAGCATTGGAAT	TGTAGGGCATCTGAGAACGAG
PPARγ2	TCAGTGGAGACCGCCCA	TCTGAGGTCTGTCATTTTCTGGAG
αP2	GAAGTAGGAGTGGGCTTTGC	CATGACGCATTCCACCACCA
Osteocalcin	TGAAGAGACCCAGGCGCTA	GATGTGGTCAGCCAACTCGTC
IL-6	ATGTAGCCGCCCCACACAGA	GCATCCATCTTTTTCAGCCATC
IL-6R	CATGCTTTGGGTGGAATGGAC	CATCAACAGGAAGTTGGTCCC
IL-17	GTGAAGGCAGGAATCACAATC	ACCAGGATCTCTTGCTGGAT
IL-17R	GATGACAGCTGGATTCACC	CTCATATTCCTGGTCAGGG
β-actin	TCAAGATCATTGCTCCTCCTG	CTGCTTGCTGATCCACATCTG
