# Supplementary material for: Tumor-Like Stem Cells Derived from Human Keloid Are Governed by the Inflammatory Niche Driven by IL-17/IL-6 Axis
Source: PLoS One. 2009 Nov 11;4(11):e7798. doi: 10.1371/journal.pone.0007798 (PMC2771422; doi:10.1371/journal.pone.0007798)
Supplement: Table S3 — Quantification of cytokine antibody array (0.05 MB PDF) [file pone.0007798.s004.pdf]

Table S3: Quantification of cytokine antibody array*

Cytokines	Normal Skin	Keloid	Keloid/Normal	Cytokines	Normal Skin	Keloid	Keloid/Normal
ENA-78	763.50	1,051.12	1.33	MCP-1	5,019.00	7,159.57	1.43
G-CSF	173.50	241.72	1.39	MCP-2	142.66	182.67	1.28
GM-CSF	153.00	187.47	1.23	MCP-3	58.43	45.85	0.78
GRO	2,932.50	3,056.68	1.05	M-CSF	225.50	285.40	1.26
GRO-alpha	1,164.00	2,349.68	2.02	MDC	133.50	242.20	1.81
I-309	102.00	118.34	1.16	MIG	141.29	131.30	0.93
IL-1alpha	167.23	186.99	1.12	MIP-1-delta	193.00	411.66	2.13
IL-1beta	68.12	133.70	1.96	RANTES	108.00	279.16	2.58
IL-2	88.00	118.34	1.34	SCF	250.28	565.29	2.25
IL-3	89.50	107.30	1.20	SDF-1	73.22	63.61	0.86
IL-4	118.00	145.70	1.23	TARC	0.00	0.00	
IL-5	386.00	528.80	1.37	TGF-beta 1	566.87	1,523.91	2.69
IL-6	21,616.50	62,790.20	2.91	TNF-alpha	313.57	714.63	2.28
IL-7	1,141.50	1,780.35	1.56	TNF-beta	417.50	575.37	1.38
IL-8	24,054.00	25,108.55	1.04	EGF	231.00	324.77	1.41
IL-10	528.50	826.45	1.56	IGF-1	291.50	374.22	1.28
IL12-p40	134.87	154.34	1.14	Angiogenin	494.00	3,361.25	6.81
IL-13	20.81	27.60	1.32	Oncostatin M	111.00	166.83	1.50
IL-15	165.37	187.47	1.13	TPO	101.00	136.10	1.35
IFN-γ	90.23	120.26	1.33	VEGF	541.00	1755.83	3.24
Leptin	83.42	76.57	0.92	PDGF-BB	285.12	580.89	2.04

*Cytokine profiling of keloid and matched normal skin tissue lysates and differential expression of candidate gene products by Human Cytokine Antibody Array.
