# Supplementary material for: Tumor-Like Stem Cells Derived from Human Keloid Are Governed by the Inflammatory Niche Driven by IL-17/IL-6 Axis
Source: PLoS One. 2009 Nov 11;4(11):e7798. doi: 10.1371/journal.pone.0007798 (PMC2771422; doi:10.1371/journal.pone.0007798)
Supplement: Table S4 — Transplantation with a range of number of dermal stem cells (0.01 MB PDF) [file pone.0007798.s005.pdf]

**Table S4: Transplantation with a range of number of dermal stem cells***

| Cell Number | $1x10^2$ | $1x10^3$ | $1x10^4$ | $1x10^5$ | $5x10^5$ | $1x10^6$ | $2x10^6$ |
|---|---|---|---|---|---|---|---|
| SKPs | - | - | - | - | 1/3 | 3/3 | 3/3 |
| KPCs | - | - | - | 2/3 | 3/3 | 3/3 | 3/3 |
