# Supplementary figures and images for: Tumor-Like Stem Cells Derived from Human Keloid Are Governed by the Inflammatory Niche Driven by IL-17/IL-6 Axis
Source: PLoS One. 2009 Nov 11;4(11):e7798. doi: 10.1371/journal.pone.0007798 (PMC2771422; doi:10.1371/journal.pone.0007798)

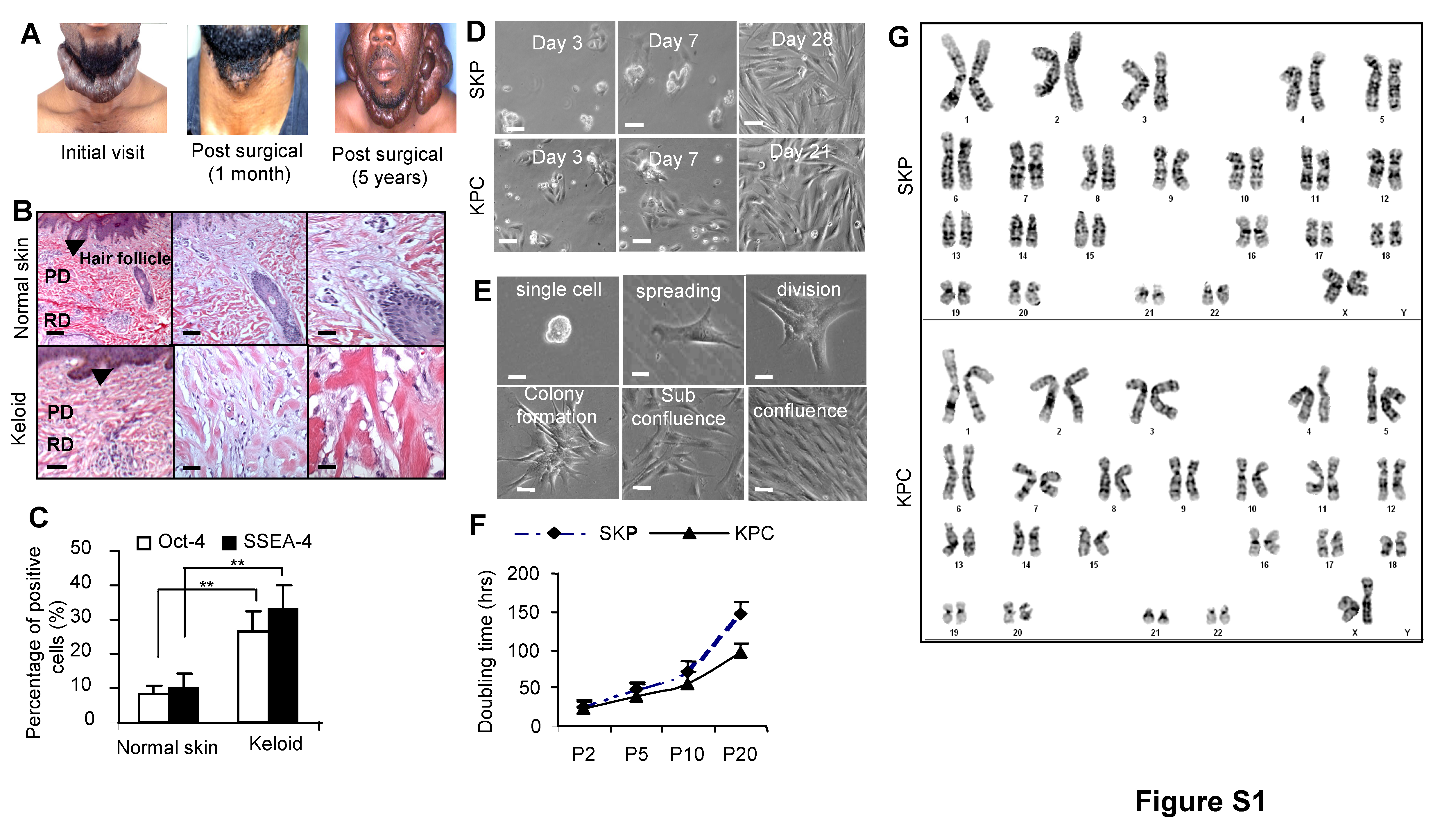

Supplement: Figure S1 — Isolation of precursor cells from keloid tissues. (A) Keloid scar displays benign tumor phenotype in terms of growth and recurrence. (B) H&E histological stain of keloid tissues and the matched peripheral normal skins. PD: papillary dermis; RD: reticular dermis. Scale bars, 50 µm. (C) Semi-quantification of immunohistochemical staining of Oct-4 and SSEA-4 in keloid and the matched normal skin (Fig. 1A) as described in Materials & Methods. **P<0.01. (D) Colony formation of stem cells derived from keloid (KPCs) and normal skin (SKPs). (E) Subcloning and culture of mesenchymal stem cells from keloids (KPCs) in α-MEM medium supplemented with 10% FBS, 1 x NEAA (non-essential amino acid) and antibiotics. Scale bars, 100 µm. (F) Determination of doubling time of KPCs and SKPs as described in Materials and Methods (mean±SEM). (G) Karyotyping of the SKP and KPC clones at passage 10. The results are representative of at least five independent experiments. (7.14 MB TIF) [file pone.0007798.s006.tif]

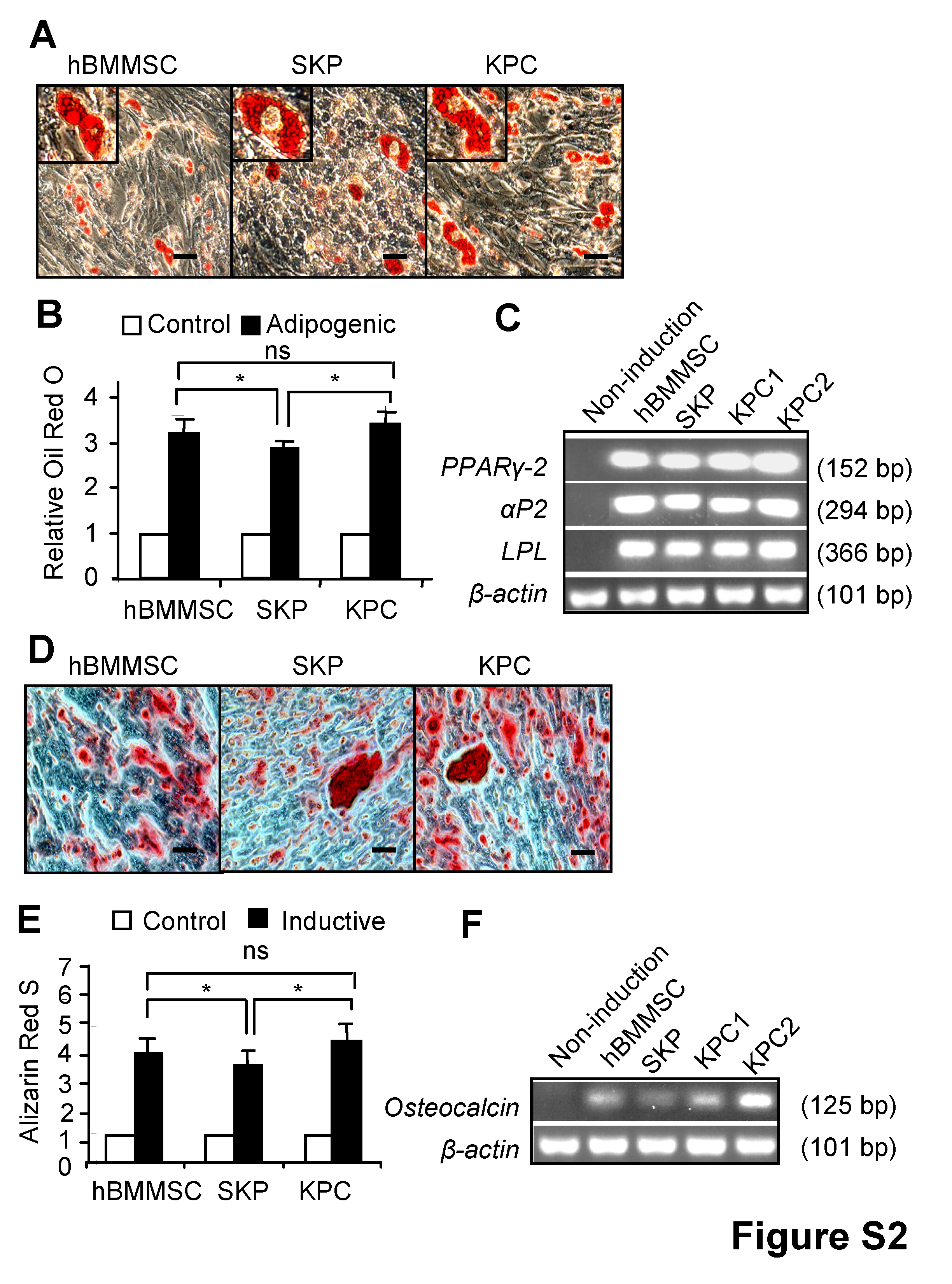

Supplement: Figure S2 — Multipotent differentiation of keloid derived precursor cells (KPCs). (A–C) Adipogenic differentiation of SKP or KPC as determined by Oil Red O staining (A and B) and RT-PCR analysis of specific adipocyte genes (C). (D–F) Osteogenic differentiation as determined by Alizarin Red S staining (D and E) and RT-PCR analysis of osteocalcin gene (F). Human bone marrow mesenchymal stem cells (hBMMSCs) were used as positive controls whereas KPCs culturing under normal growth medium were served as non-induction control. Scale bars, 50 µm. Data are representative of at least five independent experiments using KPCs and the matched SKPs from 5 different patient donors (mean±SEM). * P<0.05; ns, no significance. (5.20 MB TIF) [file pone.0007798.s007.tif]

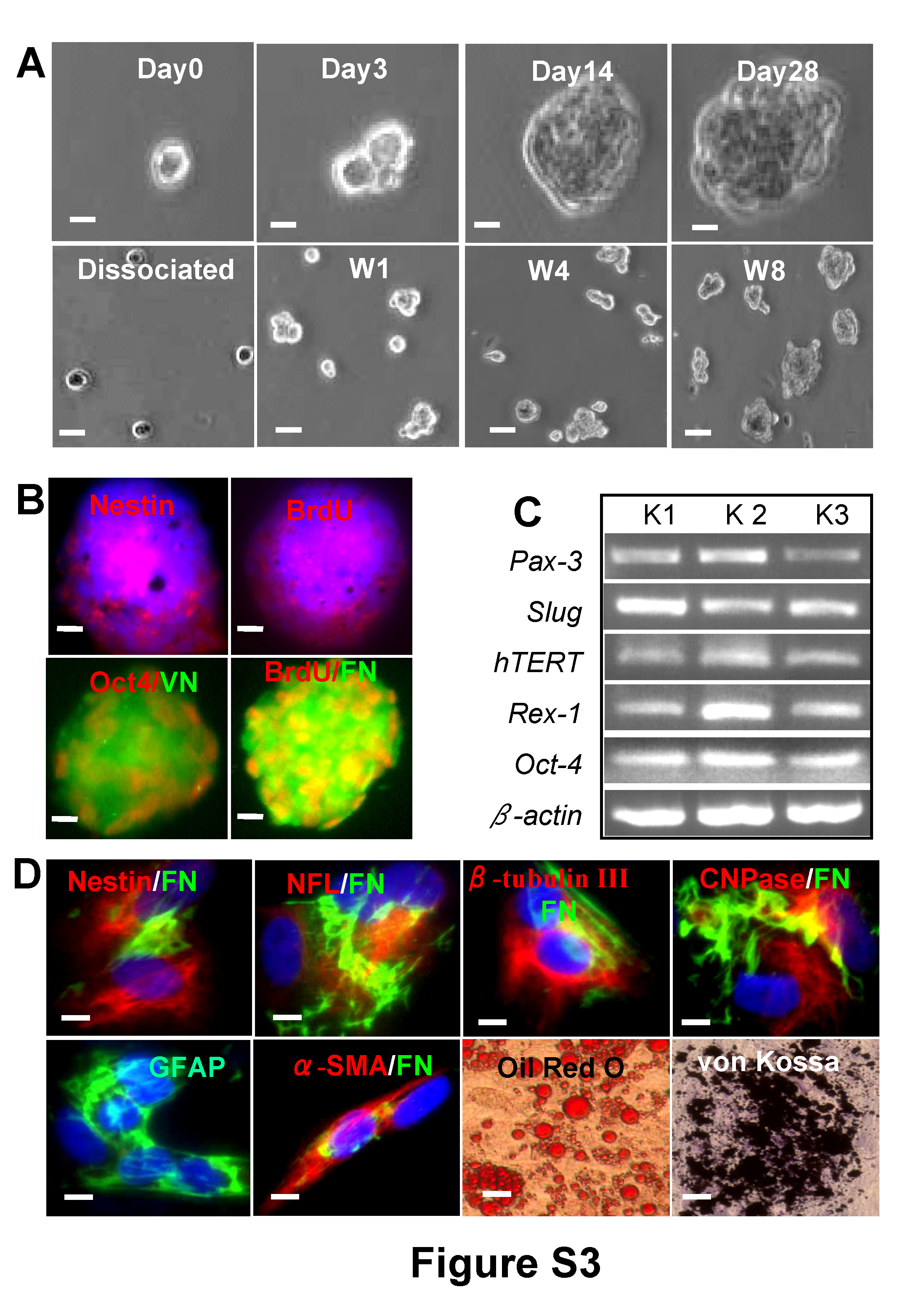

Supplement: Figure S3 — Sphere-colony formation of KPCs. (A) Subcloning and expansion of sphere-colonies derived from keloids in DMEM-LG/F12 (3∶1) supplemented with 40 ng/mL FGF-2, 20 ng/mL EGF, B27 and antibiotics. Scale bars, 100 µm. (B and C) Expression of stem cell markers and BrdU incorporation by keloid-derived sphere colonies (K1∼K3) as determined by immunofluorescence staining (B) and RT-PCR analysis (C). Scale bars, 50 µm. (D) Multipotent differentiation of keloid-derived sphere colonies into different lineages of neural cells, adipocytes, and osteocytes as determined by immunofluorescence staining with specific neural cell markers, Oil Red O and von Kossa staining. Scale bars, 50 µm. The results are representative of at lease five independent experiments. (4.37 MB TIF) [file pone.0007798.s008.tif]

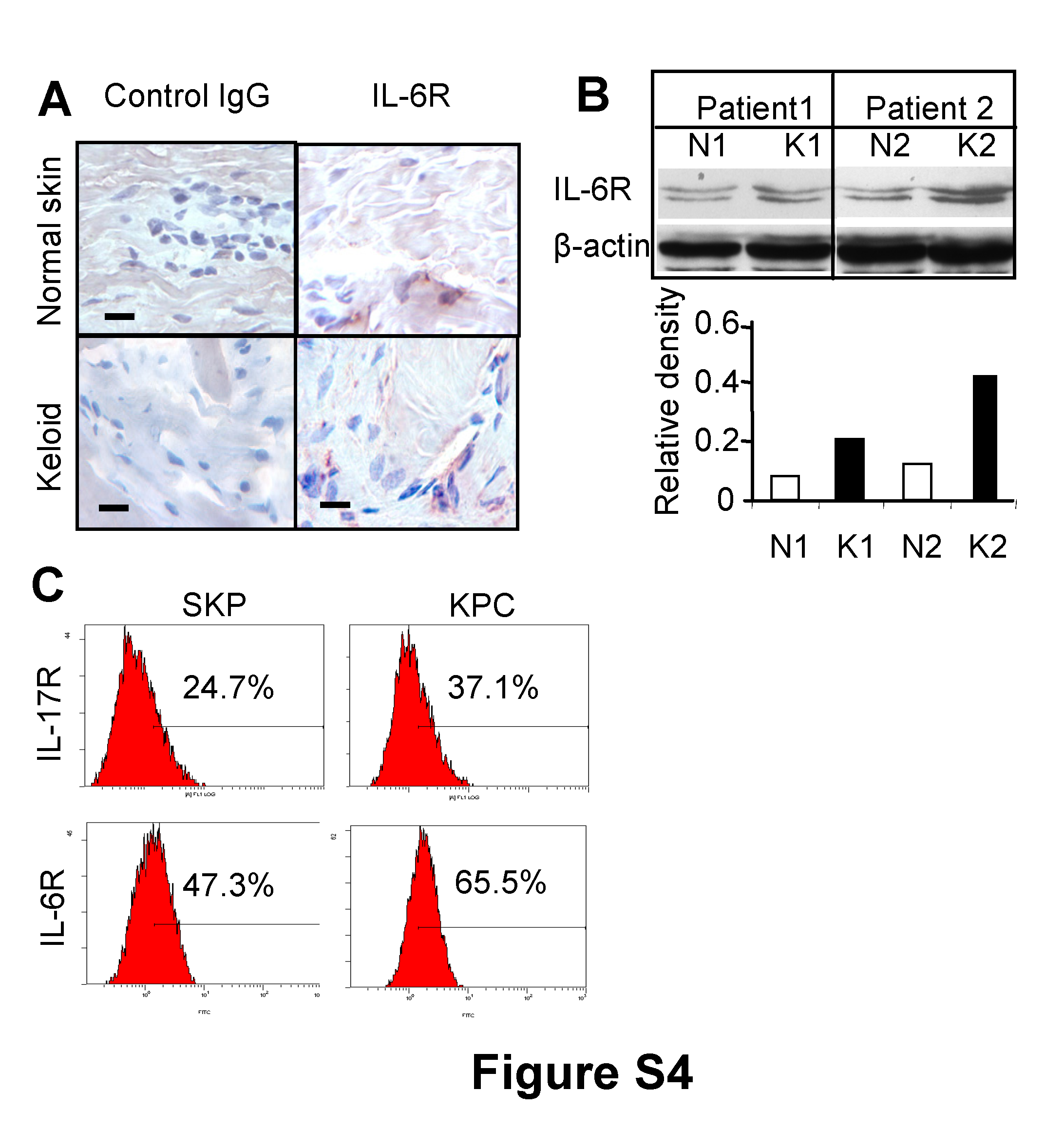

Supplement: Figure S4 — Expression of IL-6 receptor (IL-6R) in keloids (K1, K2) and matched normal skins (N1, N2). (A) Paraffin-embedded sections of keloid and the matched normal skin were immunostained with a specific antibody for human IL-6R or an isotype-matched control IgG. Scale bars, 50 µm. (B) Western blot analysis of IL-6R in tissue lysates. (C) Flow cytometric analysis of IL-6R and IL-17R expression in cultured SKPs or KPCs. The results are representative of five independent experiments. (1.72 MB TIF) [file pone.0007798.s009.tif]

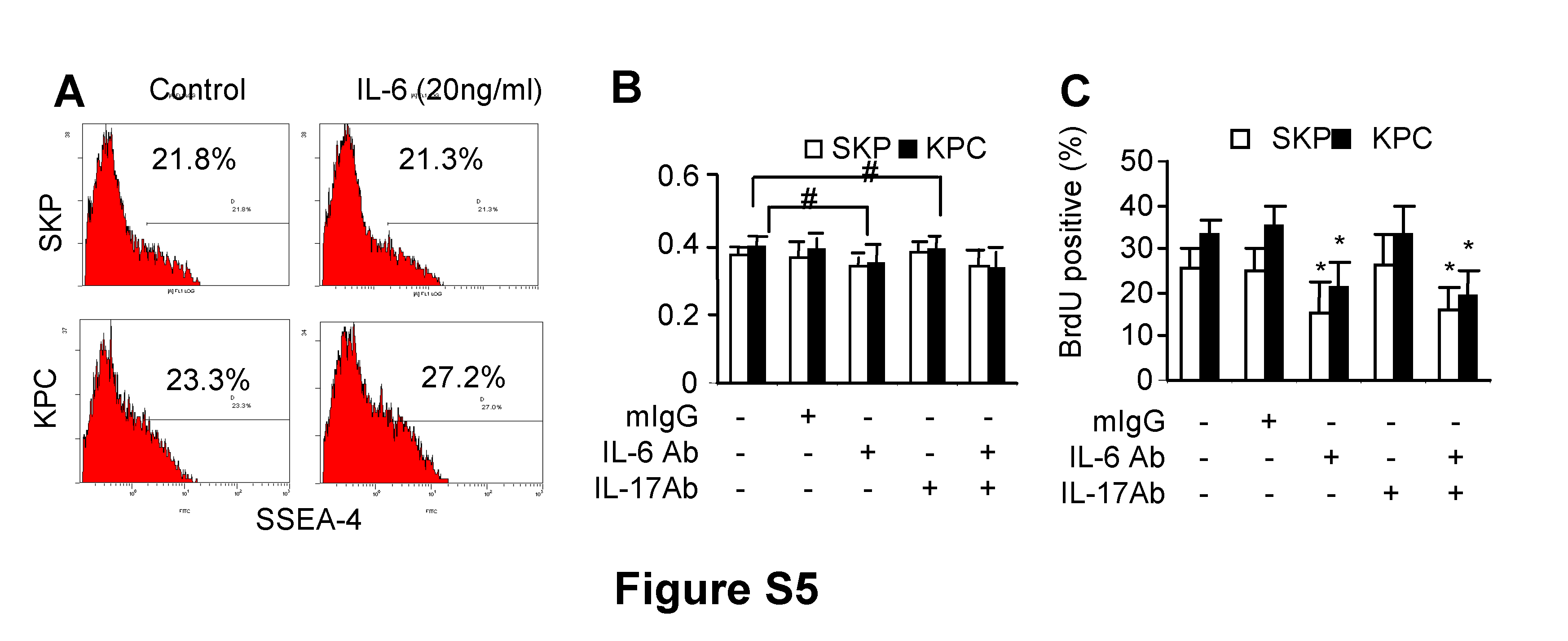

Supplement: Figure S5 — Effect of IL-6 on the expression of SSEA-4 and BrdU incorporation in SKPs and KPCs. (A) Cells were stimulated with 20 ng/ml IL-6 for 24 h followed by immunostained with antibodies for SSEA-4 and FITC-conjugated secondary antibody and analyzed by flow cytometry. (B and C) Effects of IL-6 and IL-17 neutralizing antibodies on cell viability and proliferation in SKPs and KPCs. Cells were treated for 24 h with 5 µg/ml of neutralizing antibodies for human IL-6, or IL-17, or both in the absence of IL-6 and IL-17, whereby an isotype-matched normal mice antibody (mIgG) was used as negative controls. Cell viability and proliferation were determined by MTT (B) and BrdU incorporation assay (C), respectively. The results are representative of at least five independent experiments (mean±SEM). #, no significant difference; * P<0.05, as compared with non-treatment control. (0.45 MB TIF) [file pone.0007798.s010.tif]

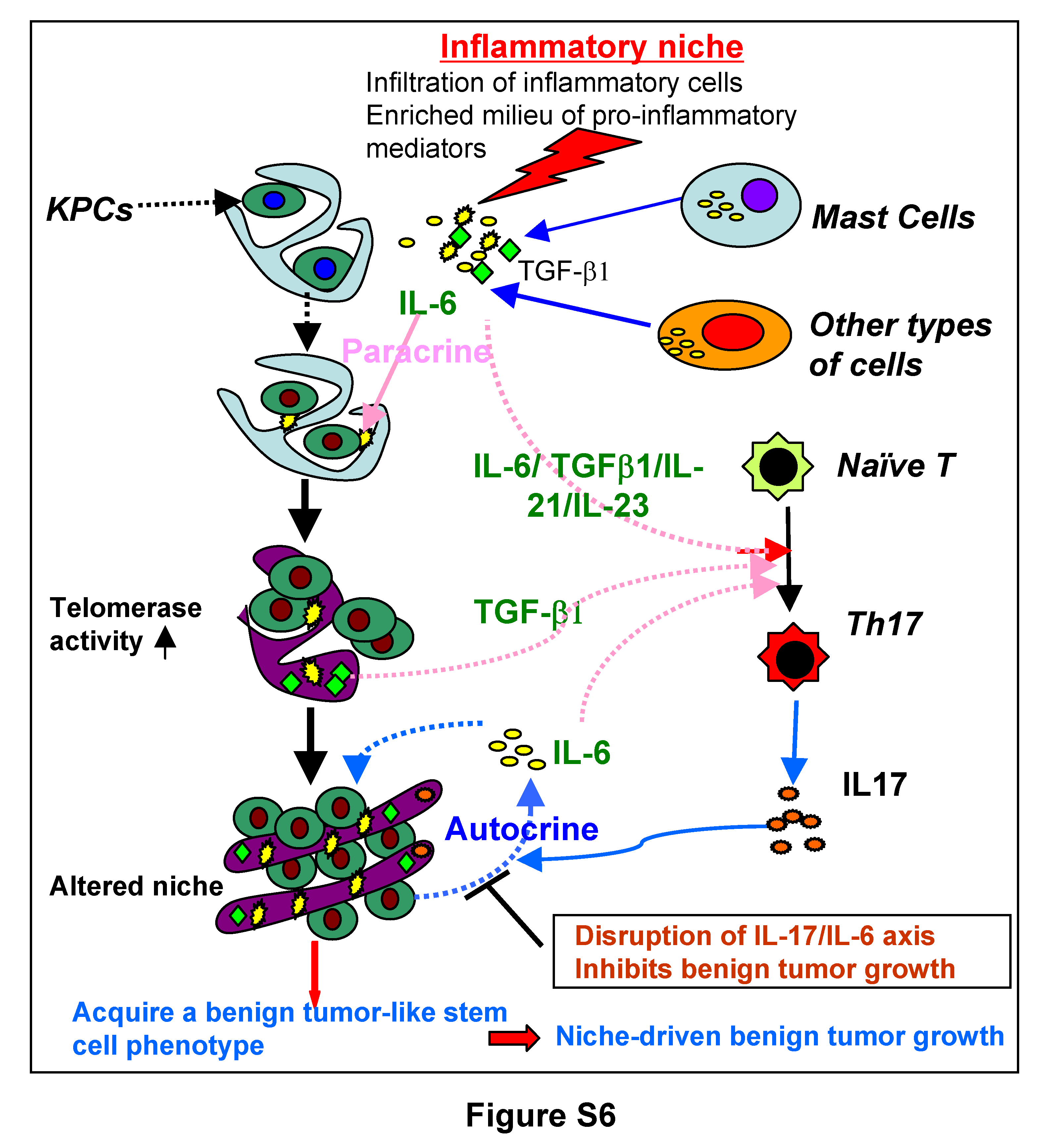

Supplement: Figure S6 — Schemed inflammatory niche-driven benign tumor growth model. Under the chronic inflammatory microenvironment, keloid-derived precursor cells (KPCs) are persistently interacted with inflammatory cells and stimulated by enriched milieu of pro-inflammatory mediators, specifically IL-6, and then acquired a benign tumor-like stem cell phenotype characterized by moderately increased telomerase activity, and consequently, an increased proliferative capacity, whereby the increased IL-17 continuously drives this process by augmenting the production of IL-6 by KPCs, thus leading to the overgrowth of keloid benign tumor. (0.87 MB TIF) [file pone.0007798.s011.tif]
